# Mechanistic Insights into the Potentiodynamic Electrosynthesis of PEDOT Thin Films at a Polarizable Liquid|Liquid Interface

**DOI:** 10.1021/jacs.4c09638

**Published:** 2024-10-09

**Authors:** Rob A. Lehane, Alonso Gamero-Quijano, José A. Manzanares, Micheál D. Scanlon

**Affiliations:** a The Bernal Institute and Department of Chemical Sciences, School of Natural Sciences, University of Limerick (UL), Limerick V94 T9PX, Ireland; b Instituto de Catálisis y Petroleoquímica − Consejo Superior de Investigaciones Científicas (ICP − CSIC), Calle de Marie Curie 2, Madrid 28049, Spain; c Department of Thermodynamics, Faculty of Physics, University of Valencia, c/Dr. Moliner, 50, Burjasot, Valencia E-46100, Spain

## Abstract

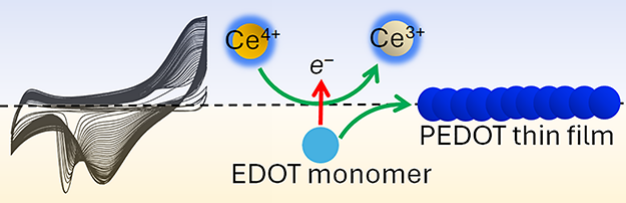

Conducting polymer (CP) thin films find widespread use, for example in bioelectronic, energy harvesting and storage, and drug delivery technology. Electrosynthesis at a polarizable liquid|liquid interface using an aqueous oxidant and organic soluble monomer provides a route to free-standing and scalable CP thin films, such as poly(3,4-ethylenedioxythiophene) (PEDOT), in a single step at ambient conditions. Here, using the potentiodynamic technique of cyclic voltammetry, interfacial electrosynthesis involving ion exchange, electron transfer, and proton adsorption charge transfer processes is shown to be mechanistically distinct from CP electropolymerization at a solid electrode|electrolyte interface. During interfacial electrosynthesis, the applied interfacial Galvani potential difference controls the interfacial concentration of the oxidant, but not the CP redox state. Nevertheless, typical CP electropolymerization electrochemical behaviors, such as steady charge accumulation with each successive cycle and the appearance of a nucleation loop, were observed. By combining (spectro)electrochemical measurements and theoretical models, this work identifies the underlying mechanistic origin of each feature on the cyclic voltammograms (CVs) due to charge accumulated from Faradaic and capacitive processes as the PEDOT thin film grows. To prevent overoxidation during interfacial electrosynthesis with a powerful cerium aqueous oxidant, scan rates in excess 25 mV·s^–1^ were optimal. The experimental methodology and theoretical models outlined in this article provide a broadly generic framework to understand evolving CVs during interfacial electrosynthesis using any suitable oxidant/monomer combination.

## Introduction

Conducting polymers (CPs) constitute a class of materials with unique mechanical, optoelectronic, redox, and photovoltaic properties.^1^ CPs find widespread applications in bioelectronic (*e.g.,* neural interfaces, organic electrochemical transistors, electronic skin),^2−5^ energy harvesting and storage (*e.g.,* batteries, supercapacitors, organic solar cells),^6−8^ electrochromic,^9^ (bio)sensor,^10^ drug delivery,^11^ and soft robotic^12^ technology. In a majority of these technologies, the CPs are incorporated as two-dimensional (2D) thin films to take advantage of their light, flexible, and transparent nature.^13^

Liquid|liquid (L|L) interfaces provide a simple, template-free, and cost-effective route to 2D thin film synthesis.^14−17^ Certain L|L interfaces, known as interfaces between two immiscible electrolyte solutions (ITIES),^18−20^ are polarizable and enable electrochemical control over the interfacial synthesis of thin films. The foundational work on electrosynthesis of CPs, *e.g.,* poly(thiophene), poly(terthiophene), and various derivatives of poly(pyrrole), and their nanocomposites with metallic nanoparticles or carbon nanotubes at polarizable L|L interfaces was carried out by the groups of Cunnane et al.,^21−30^ Mareček et al.,^31^ and Dryfe et al.^32^ CP nanocomposites with metallic nanoparticles were also electrosynthesized at a miniaturized aqueous|1,2-dichloroethane interface by Stockmann’s group^33−35^ and a miniaturized aqueous|hydrophobic ionic liquid interface by Nishi et al.^36^ The basic concept is that, through polarization of the L|L interface, electrochemical control over the kinetics of interfacial electron transfer (IET) between aqueous soluble oxidant and organic soluble monomer redox couples is achievable. Recently, we reported an advance in the use of electrosynthesis at a polarizable aqueous|α,α,α-trifluorotoluene (TFT) interface to prepare free-standing, additive-free, reproducible, easily transferrable, scalable, and biocompatible poly(3,4-ethylenedioxythiophene) (PEDOT) thin films in a single step at ambient conditions using aqueous Ce^4+^ and organic soluble EDOT.^37^

Our study of the electrosynthesis of PEDOT thin films by double potential step chronoamperometry (DPSCA) at a polarizable aqueous|TFT interface demonstrated that the IET step between Ce^4+^ and EDOT to form cationic EDOT oligomers occurs at a positive, externally applied interfacial Galvani potential difference Δ_o_^w^ϕ, while interfacial adsorption of the oligomers occurs at a more negative Δ_o_^w^ϕ.^37^ As a consequence, potentiodynamic or multistep potentiostatic techniques are favored over single-step potentiostatic electrochemical techniques.

In this work, we significantly deepen our understanding of the mechanism of electrosynthesis at a polarizable aqueous|TFT interface by preparing PEDOT thin films using the classic potentiodynamic technique of cyclic voltammetry (CV). For the first time, we show that features typical of electropolymerization of CPs by repetitive CV cycling at solid electrode|electrolyte interfaces, such as a steady growth of the charge accumulated with each successive CV cycle and the observation of a nucleation loop during the initial CV cycles, are replicated during CP thin film electrosynthesis at a polarizable L|L interface. The cyclic voltammograms (CVs) display three distinct features at different applied potentials that evolve with repetitive cycling and are attributed to IET between Ce^4+^ and EDOT, interfacial adsorption of the EDOT oligomers with an associated anion exchange process, and reversible proton adsorption/desorption on the growing PEDOT thin film, respectively. The doping state of the PEDOT thin film deposited at the aqueous|TFT interface is not controlled with the applied Δ_o_^w^ϕ during interfacial electrosynthesis. This is in stark contrast to CP electropolymerization at solid electrode|electrolyte interfaces, where the applied potential modulates the redox state (*p-* or *n*-doping) of the electrodeposited film. Theoretical models are provided to interpret each of these voltammetric features, with the Faradaic or capacitive nature of each charge transfer process identified. The influence of the scan rate during cycling on PEDOT interfacial electrosynthesis is explored and an optimal scan rate range identified to prevent overoxidation.

## Results and Discussion

### Interfacial Electrosynthesis by Repetitive CV Cycling: Initial Observations

**Figure 1:**
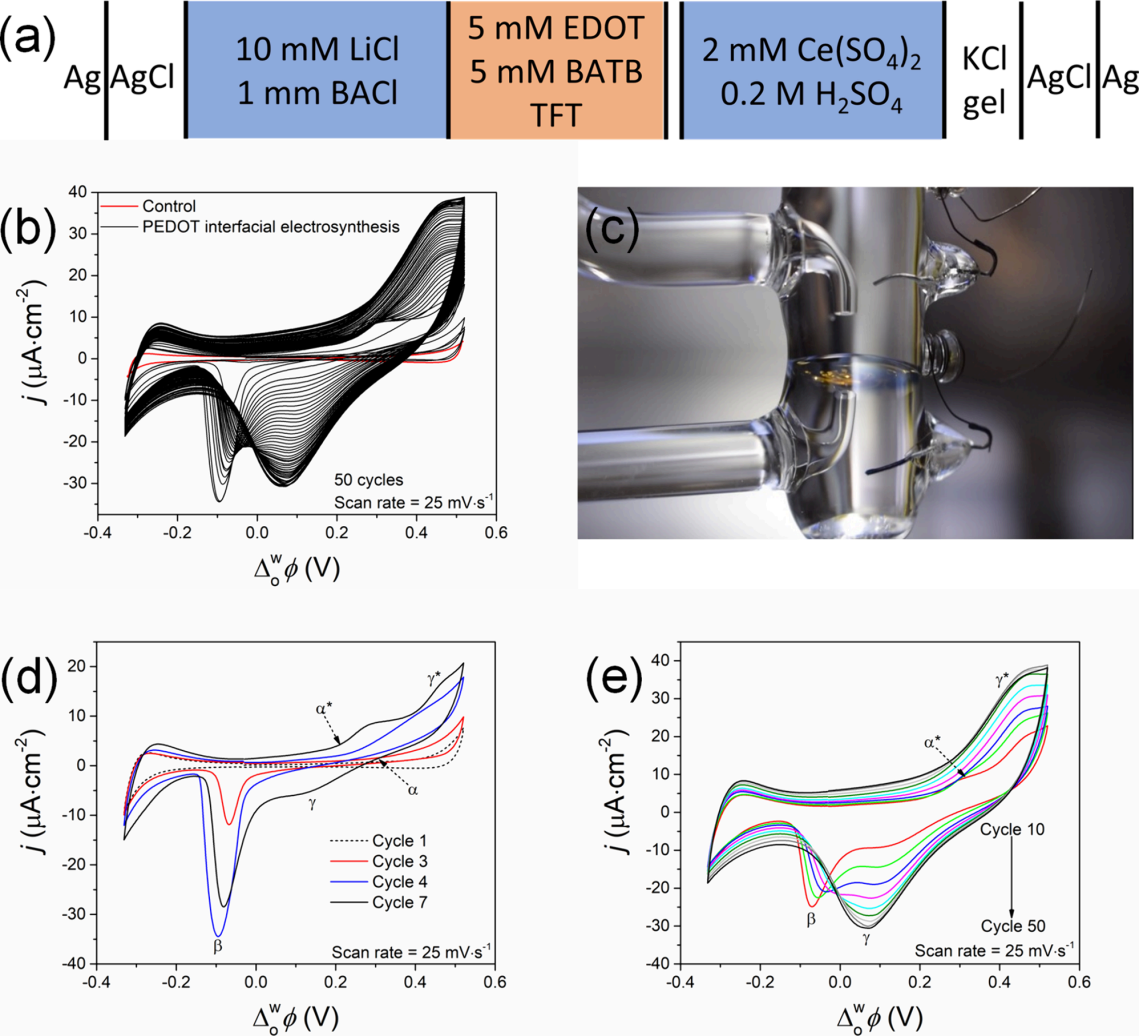
Electrosynthesis of a PEDOT thin film by repetitive cyclic voltammetry (CV) cycling at a polarizable aqueous|α,α,α-trifluorotoluene (TFT) interface. (a) Electrochemical cell configuration of the four-electrode electrochemical cell employed. In the organic reference solution, BACl is bis(triphenylphosphoranylidene)ammonium chloride, while BATB is the organic electrolyte bis(triphenylphosphoranylidene)ammonium tetrakis(pentafluorophenyl)borate. In this four-electrode configuration, the Pt electrode in the organic phase and Ag/AgCl electrode in the organic reference solution (1 mM BACl and 10 mM LiCl) were connected to the counter and reference terminals, respectively, while the Pt and Ag/AgCl/KCl gel electrodes in the aqueous phase were connected to the working and sensing terminals, respectively. (b) Cyclic voltammograms (CVs) were recorded, 50 repetitive CV cycles in total (black CVs), using the electrochemical cell configuration described in (a). For control experiments, the Ce(SO_4_)_2_ concentration was 0 mM (red CV). (c) Image of the blue PEDOT thin film formed at the polarizable liquid|liquid interface after 50 CVs. (d) Selected cycles during the initial stages of interfacial electrosynthesis at a bare interface, i.e., cycles 1 to 7, with three distinct electrochemical features (labeled α/α*, β, and γ/γ*, respectively) that evolve with cycling at different applied . (e) Selected cycles after EDOT oligomers have adsorbed at the interface and the PEDOT thin film is growing, i.e., every fifth cycle is shown from cycles 10 (red CV) to 50 (black CV). All CVs were recorded under ambient, aerobic conditions at a scan rate of 25 mV·s^–1^.

To initiate and control the thermodynamically nonspontaneous interfacial electrosynthesis of PEDOT using a Ce^4+^ oxidant (in the form of Ce(SO_4_)_2_), the aqueous|TFT interface must be polarized using a potentiostat in conjunction with a four-electrode electrochemical cell (see Section S1 and Figure S1).^37^ Herein, the external electrochemical driving force, the interfacial Galvani potential difference , was applied by CV using the electrochemical cell configuration described in Figure 1a. After 50 repetitive CV cycles (Figure 1b), a homogeneous blue PEDOT thin film can be seen coating the polarizable L|L interface (Figure 1c). The 50 CVs shown in Figure 1b (black CVs) display numerous continually evolving features with repetitive cycling at 25 mV·s^–1^. Control CVs obtained in the absence of Ce^4+^ show none of these characteristic features associated with PEDOT interfacial electrosynthesis (Figure 1b, red CVs). The charge accumulated at the L|L interface grows steadily with each successive cycle during interfacial electrosynthesis, in a manner analogous to that of conventional CP electropolymerization at a solid electrode|electrolyte interface, for example of PEDOT, poly(terthiophene), or poly(aniline).^27,38,39^ This increase in the charge accumulated is attributed to a combination of Faradaic and capacitive currents that arise due to the growth of the PEDOT thin film at the L|L interface, i.e., due to processes entirely separate from those that occur during electropolymerization at a solid electrode|electrolyte interface. The structure of the electrical double layer (EDL) at a polarizable L|L interface differs from that at a solid electrode|electrolyte interface in that it consists of two back-to-back aqueous and organic diffuse layers.^20^ Thus, in the presence of a growing PEDOT thin film, capacitive currents in particular may arise from processes occurring simultaneously in the EDLs at the aqueous|PEDOT and PEDOT|organic interfaces, respectively.

**Figure 2:**
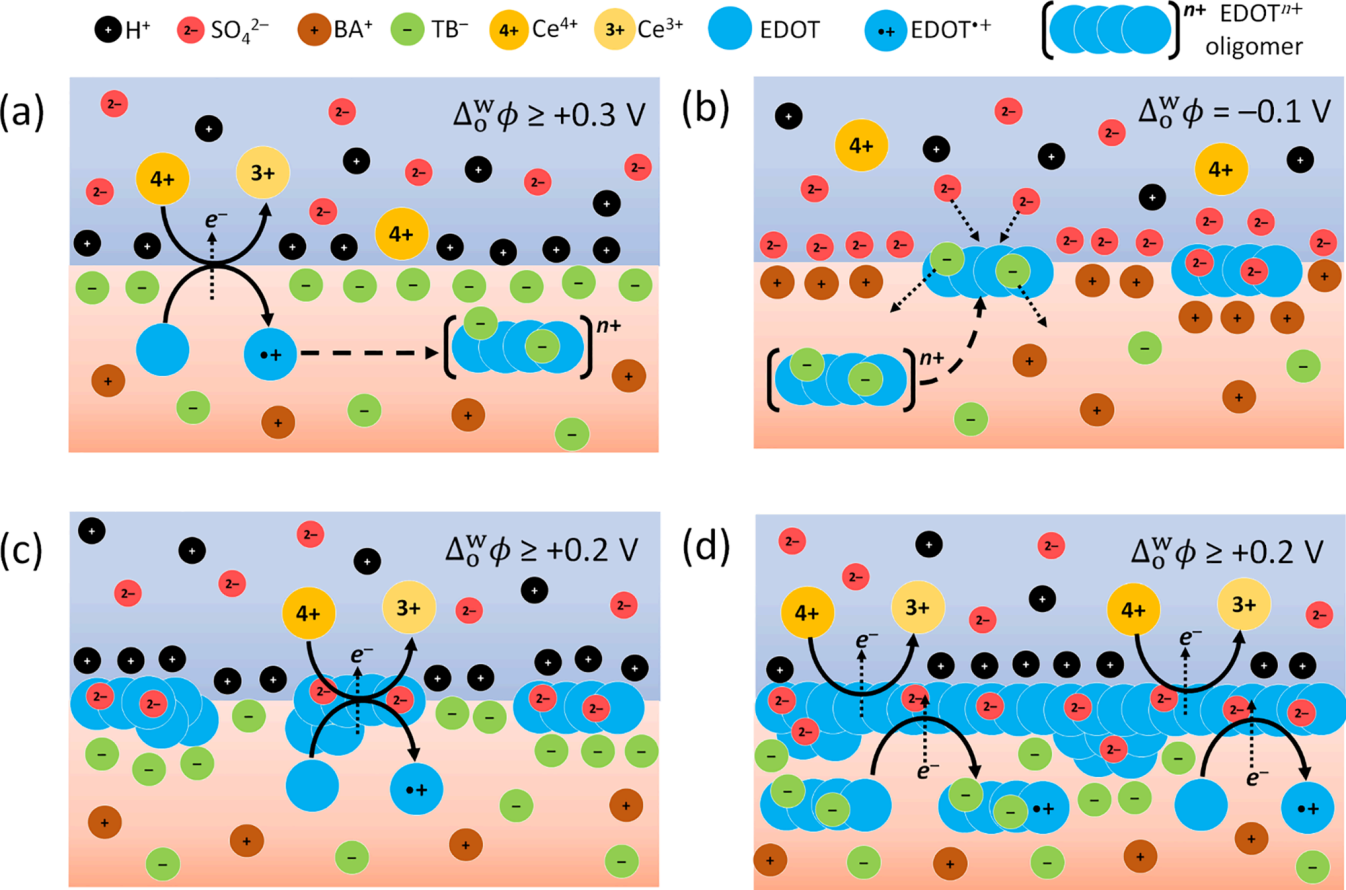
Schematics of the progress of PEDOT interfacial electrosynthesis at a polarizable L|L interface with repetitive CV cycling. (a) Interfacial electron transfer (IET) reaction between Ce^4+^ and EDOT monomers at the bare L|L interface, yielding EDOT^•+^ species in the organic side of the electrical double layer (EDL), at an applied  ≥ 0.3 V. (b) Anion exchange process at an applied Δ_o_^w^ϕ of −0.1 V. As EDOT oligomers adsorb at the bare L|L interface during PEDOT thin film electrosynthesis, weakly coordinating organic soluble TB^–^ are exchanged by aqueous SO_4_^2–^. (c) Adsorbed EDOT oligomers acting as floating interfacial bipolar-like electrodes and catalyzing the IET reaction between Ce^4+^ and EDOT monomers. (d) Simultaneous oxidation of EDOT monomers and oligomers by the electrochemically polarizable PEDOT thin film during later CV cycles when the PEDOT thin film coats the entirety of the L|L interface.

Individual CVs are highlighted in Figures 1d,e to show the evolution of three distinct electrochemical features (labeled α/α*, β, and γ/γ*, respectively) at different applied  as interfacial electrosynthesis proceeds with repetitive CV cycling at 25 mV·s^–1^. The first feature (labeled α), observed during the forward scan (scanning from negative to positive ) of the third cycle in Figure 1d, is a slight increase in current with an onset approaching +0.3 V that increases in magnitude significantly by the fourth cycle. This irreversible charge transfer process takes place over the first four cycles and is attributed to IET between Ce^4+^ and EDOT monomers at the bare L|L interface, as shown schematically in Figure 2a.

The second feature (labeled β), observed on the backward scan, is a sharp negative peak that occurs at ca. –0.1 V. The shape of the peak, in terms of the absence of a diffusional tail, and a sharp increase in intensity suggest a type of adsorption or surface process at the polarizable L|L interface.^40^ We attribute this feature to the interfacial adsorption of EDOT oligomers at the bare L|L interface. The EDOT oligomers are cationic in nature, as demonstrated *vide infra*, and upon adsorption are involved in an anion exchange process where the weakly coordinating organic soluble tetrakis(pentafluorophenyl)borate anions (TB^–^) anions are exchanged by aqueous sulfate anions (SO_4_^2–^) (see Figure 2b). This interpretation is supported by a previous X-ray photoelectron spectroscopy study where PEDOT thin films formed by interfacial electrosynthesis were found to be exclusively doped by SO_4_^2–^, with no traces of TB^–^ found.^37^ The electrostatic charge on the cationic EDOT oligomers is increasingly compensated by SO_4_^2–^ anions, as the applied  is scanned more negative than the potential of zero charge (PZC), giving rise to the sharp negative peak (modeled *vide infra*). The magnitude of this peak rapidly increases until the fourth cycle (Figure 1d), after which the peak abruptly begins to decrease in magnitude and shift to a slightly more positive applied  with subsequent cycles (Figures 1d,e). The latter signifies a rapid loss of interfacial anion exchange sites for the EDOT oligomers at the bare L|L interface. The CVs only show feature β on the backward scan, with no corresponding sharp positive peak seen upon reversing the scan direction. This is interpreted as an irreversibility in the anion exchange process; once the weakly coordinating TB^–^ is replaced during the initial anion exchange upon EDOT oligomer adsorption, it cannot displace SO_4_^2–^on the reverse scan.

The decrease in magnitude of the sharp negative peak labeled β at ca. −0.1 V after the fourth cycle is accompanied by the simultaneous appearance of a third feature (labeled γ) consisting of a broad negative peak centered at +0.1 V (Figure 1d). By the seventh cycle, the onset of the current during the forward scan shifts to less positive applied  (labeled α*) and is attributed to IET involving adsorbed interfacial EDOT oligomers and/or the growing PEDOT thin film via a “bipolar-like” mechanism (see Figure 2c). Additionally, a related positive peak appears at +0.45 V (labeled γ*), which is attributed to electrochemical processes occurring at the PEDOT thin film formed at the L|L interface. The feature α* becomes rapidly masked by feature γ* and disappears completely by the 15th cycle (see Figure 1e).

The appearance of γ and γ* signifies the transition from a bare to a PEDOT-coated L|L interface. Even after the interface is fully coated with an initial layer of PEDOT, electrosynthesis continues as the PEDOT thin film mediates the oxidation of both EDOT monomers and oligomers (see Figure 2d). Both γ and γ* are pH dependent (as demonstrated *vide infra*) and attributed to reversible proton adsorption/desorption on the growing PEDOT thin film. Both peaks dominate the voltammetric profile by the 25th cycle and increase in magnitude until their stabilization around the 35th cycle (Figure 1e). This is followed by very moderate increases with further cycling up to the 40th cycle, after which the voltammetric profile stabilizes until the 50th cycle. The Faradaic or capacitive origins of each of these features will be discussed in detail in the following sections.

### The Evolving Nature of the Faradaic Current due to Interfacial Electron Transfer during PEDOT Interfacial Electrosynthesis

The nature of the Faradaic currents due to IET reactions involving the aqueous Ce^4+^ oxidant change as oligomers adsorb at the bare L|L interface, and, with cycling, the PEDOT thin film grows. During the first four cycles at 25 mV·s^–1^, the positive Faradaic currents (labeled α in Figure 1d) are attributed to IET reactions between Ce^4+^ and EDOT monomers at the bare L|L interface that yield EDOT^•+^ species in the organic side of the EDL (see Figure 2a). The EDOT^•+^ species may couple with each other or another EDOT monomer to form dimers. EDOT oligomers are subsequently formed by radical coupling steps as EDOT^•+^ is continually generated by IET.

Radical coupling also leads to a release of protons in the organic side of the EDL. These protons may undergo ion transfer from the organic to aqueous phase within the full range of the Galvani polarizable potential window (PPW). Such proton transfer does not change the shape of the CVs described in Figure 1b but should generate a background current that shifts the whole CV negatively. In other words, due to proton transfer, the CVs should not be perfectly centered on zero current during interfacial electrosynthesis. However, detection of such a slight negative shift of the CVs is difficult as proton transfer is far less evident than the other charge transfer processes that occur simultaneously during CV cycling (leading to features α/α*, β, and γ/γ*).

Electroneutrality is maintained in the organic side of the EDL by electrostatic interactions between the cationic oligomers and organic TB^–^. However, as TB^–^ is a weakly coordinating anion, the EDOT oligomers maintain a net positive charge. The ionic nature of EDOT oligomers in the presence of BATB electrolyte is demonstrated by their measurable ion transfer across the polarizable L|L interface (see Figure S2).^37,41^

The interfacial Galvani potential difference  for the onset of a biphasic single-step IET reaction does not necessarily correlate with the thermodynamically determined .^42^ Instead,  may be dependent on the kinetics of the IET reaction, which are largely dictated by the potential-modulated interfacial concentration of each redox species. As described in Section S3, the latter, in turn, is dependent on  and  can be defined as
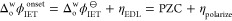
1where η_polarize_ is any additional overpotential required to polarize the L|L interface sufficiently positive of the PZC to enhance the interfacial concentration of Ce^4+^ to the level required to initiate IET with appreciable kinetics, and η_EDL_ = η_PZC_ + η_polarize_ is the overpotential of the EDL at the L|L interface.^42^

**Figure 3:**
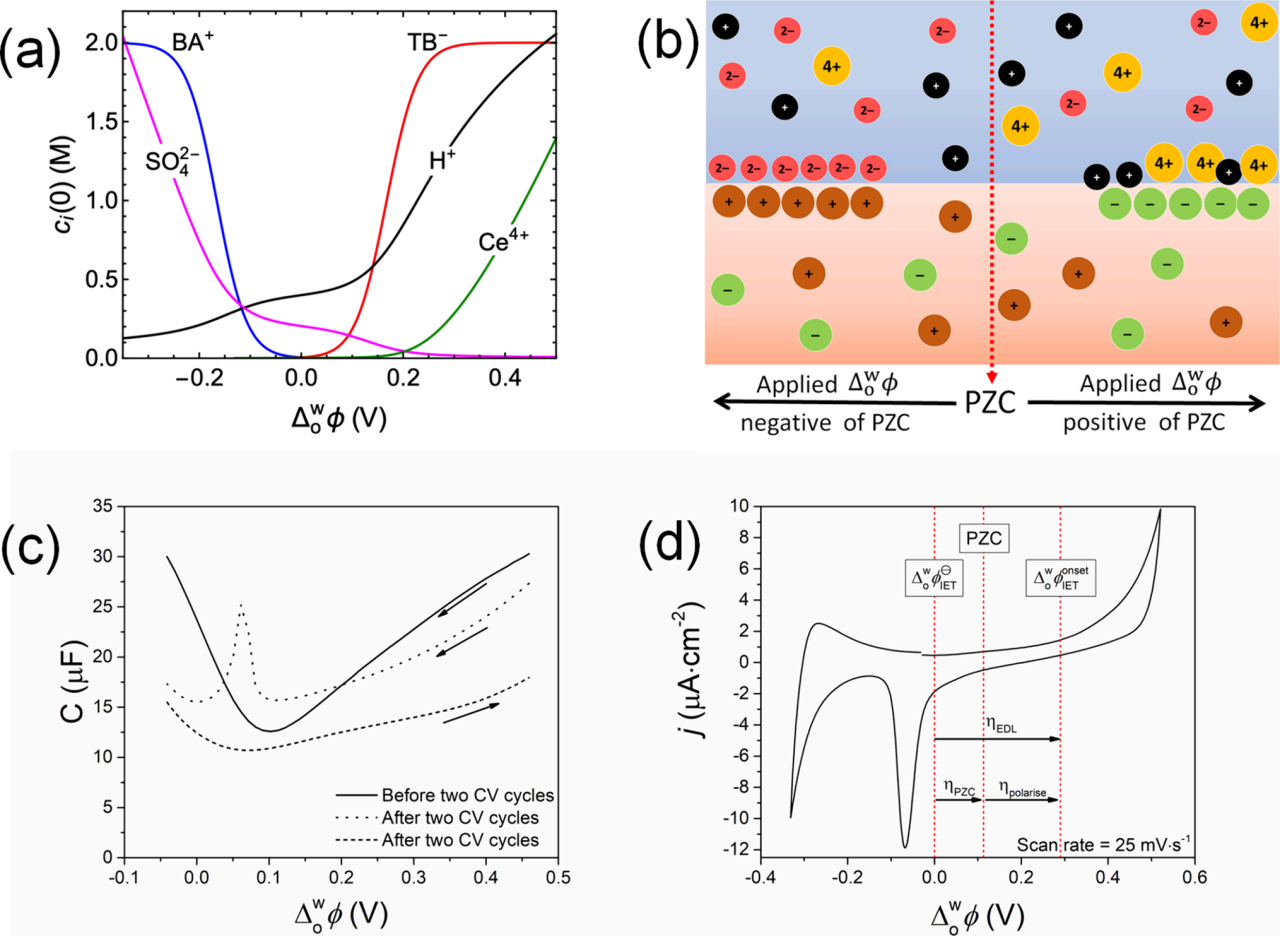
Influence of the potential-modulated distribution of ionic species on the kinetics of the IET between EDOT and Ce^4+^at a bare L|L interface. (a) Interfacial ionic concentrations *c*_*i*_(0) for the electrochemical cell configuration described in Figure 1a (in the absence of EDOT) modeled as a function of the applied  at a polarizable aqueous|TFT interface. For the model, the maximum permitted interfacial concentrations of BA^+^ and TB^–^ were defined as 2 M (see Section S4) and the potential of zero charge (PZC) was arbitrarily set at 0 V on the Galvani scale. (b) Schematic of the potential-modulated distribution of the aqueous (H^+^, Ce^4+^, and SO_4_^2–^) and organic (BA^+^ and TB^–^) ionic species as a function of the applied  relative to the PZC. (c) Differential capacitance measurements performed using the cell described in Figure 1a before (solid line) and after (dashed and dotted lines) two CV cycles at 25 mV·s^–1^. The scan direction is indicated by the arrows, and a frequency of 20 Hz with an amplitude of 10 mV was used. (d) Representation of the relative positions of the IET standard potential , the PZC, and , along with η_EDL_, η_PZC_, and η_polarize_, during the third cycle obtained at 25 mV·s^–1^ (see Figure 1d). The overpotential of the EDL at the L|L interface is η_EDL_ = η_PZC_ + η_polarize_, the sum of the required overpotential η_PZC_ beyond  to reach the PZC and any additional overpotential η_polarize_ beyond the PZC to enhance the interfacial concentration of Ce^4+^ to the level required to initiate IET with appreciable kinetics.

To investigate the correlation between the potential-modulated variation of the interfacial concentration of Ce^4+^ and , the ionic distributions of Ce^4+^, H^+^, and SO_4_^2–^ in the aqueous side of the EDL, and BA^+^ and TB^–^ in the organic side of the EDL, were calculated as a function of the applied  at a polarizable aqueous|TFT interface (Figure 3a and Figures S3–S6) by solving a modified Poisson–Boltzmann equation that takes into account the volume exclusion effect due to the BA^+^ and TB^–^ ions by using the Bikerman equation.^42−47^ A schematic describing these modeled ionic distributions is shown in Figure 3b. The model arbitrarily sets the PZC at an applied  of 0 V and uses the concentrations of each species outlined in the electrochemical cell configuration described in Figure 1a, except for the EDOT monomers. The latter were excluded as the interfacial concentration of neutral EDOT is not expected to be influenced by the applied  during interfacial electrosynthesis. The model predicts that the interfacial concentration of Ce^4+^ becomes significant when the applied  is 0.2 V more positive than the PZC (Figure 3a). This predicted value correlates precisely with the experimentally determined value of η_polarize_ () of 0.2 V. The latter was determined from the experimentally measured  of ca. 0.3 V (from the third cycle at 25 mV·s^–1^ in Figure 1d). The experimentally determined PZC prior to interfacial electrosynthesis was ca. 0.1 V; see the differential capacitance measurement in Figure 3c of the electrochemical cell configuration described in Figure 1a. For full clarity, visual representations of the relative positions of , the PZC, and , along with η_EDL_, η_PZC_, and η_polarize_, during the third cycle are provided in Figure 3d.

As Ce^4+^ is tetravalent, the interfacial concentration of Ce^4+^ is predicted to increase rapidly when η_polarize_ increases by scanning positively. For example, when η_polarize_ is 0.4 V, the interfacial concentration of Ce^4+^ is ca. 1 M, orders of magnitude higher than the 2 mM bulk concentration (Figure 3a). The robustness of the model is supported by the excellent correlation between the voltammetric features of the modeled and experimental CVs and differential capacitance curves (Figure S6). Importantly,  lies well within the limits of the available Galvani PPW, and thus the kinetics of IET between Ce^4+^ and EDOT monomers at the bare L|L interface is directly under external electrochemical control.

### (Spectro)electrochemically Monitoring Adsorption of EDOT Oligomers at the Aqueous|TFT Interface

The cationic nature of the adsorbed EDOT oligomers is proven by the negative shift of the PZC for the differential capacitance curve obtained after two CV cycles (Figure 3c, dashed line) compared to that before cycling (Figure 3c, solid line). Saturation of the interfacial region with a sufficiently high density of EDOT oligomers of the critical chain length required to induce their adsorption at the L|L interface occurs by the third cycle, as evidenced by the appearance of the sharp negative peak at ca. −0.1 V associated with the exchange between TB^–^ and SO_4_^2–^ (labeled β in Figure 1d). This anion exchange process is also seen in the differential capacitance curve obtained after two CV cycles when the scan direction is from positive to negative  (Figure 3c, dotted line). Applying an initial positive  leads to IET and oligomer formation. Then, as the differential capacitance scan progresses to more negative , these oligomers eventually adsorb giving rise to the observed spike in differential capacitance.

**Figure 4:**
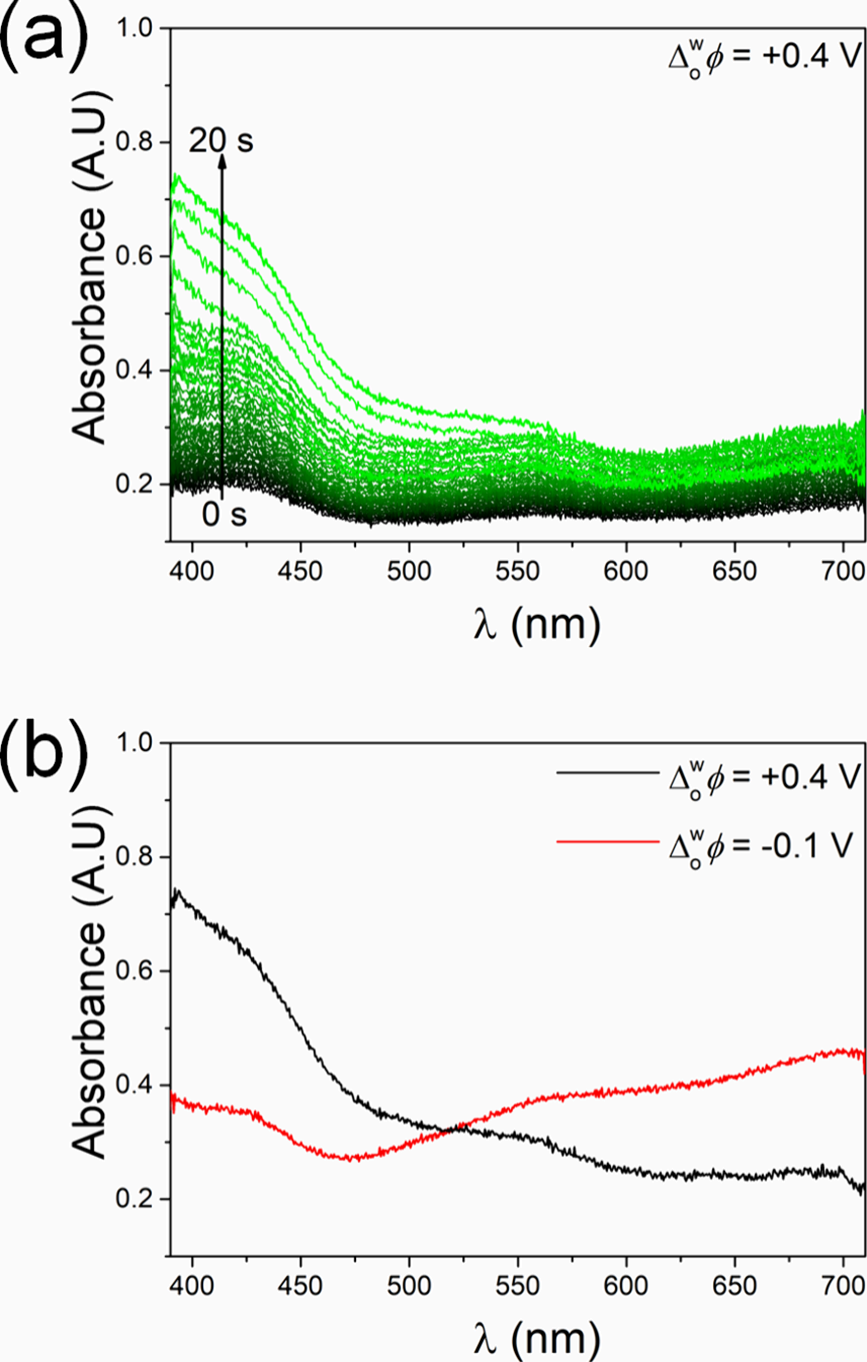
Spectroelectrochemical analysis of EDOT oligomer adsorption during interfacial electrosynthesis by *in situ* UV/vis absorption spectroscopy in total internal reflection mode (TIR-UV/vis). (a) TIR-UV/vis spectra acquired every 0.085 s for 20 s at an applied Δ_o_^w^ϕ of +0.4 V. (b) Comparison of the final TIR-UV/vis spectra recorded after 20 s at an applied Δ_o_^w^ϕ of +0.4 V and after 20 s upon stepping the applied Δ_o_^w^ϕ to −0.1 V. All spectra were recorded at ambient, aerobic conditions using the experimental setup in Figure S1b.

Further proof of the adsorption of EDOT oligomers at an applied Δ_o_^w^ϕ of −0.1 V was provided by spectroelectrochemical measurements (Figure 4). *In situ* UV/vis absorption spectra in total internal reflection mode (TIR-UV/vis) were recorded at the polarizable L|L interface using the experimental setup described in Figure S1b.^48^ The spectra were first recorded as the applied Δ_o_^w^ϕ was held at +0.4 V for 20 s. At this potential, an increase of absorbance at ∼400 nm was seen with time (Figure 4a). This signal corresponds to the formation of short EDOT oligomers in the organic side of the EDL due to IET between Ce^4+^ and EDOT monomers. Based on the position of the absorption band, it is likely that the majority of the EDOT oligomers comprise of four monomer units or less.^49,50^ When the applied Δ_o_^w^ϕ was stepped to −0.1 V for 20 s, a significant red shift in the absorbance spectrum was observed due to an increase in oligomer conjugation length (Figure 4b).^51^ The absorbance spectrum at −0.1 V is characteristic of a *p*-doped PEDOT thin film,^52^ as shown in Figure S7. It strongly indicates that the EDOT oligomers have adsorbed at the L|L interface to form the PEDOT thin film.

The spectroelectrochemical measurements in Figure 4 suggest that short EDOT oligomers are not surface active during the initial stages of interfacial electrosynthesis at an applied Δ_o_^w^ϕ of +0.4 V. The latter is close to the positive edge of the PPW, and the interfacial surface tension decreases significantly approaching both the PPW’s positive and negative edges.^53^ Conversely, an applied Δ_o_^w^ϕ of −0.1 V is comparatively further from the negative edge of the PPW leading to a relative increase in interfacial surface tension during the potential step from +0.4 to −0.1 V. The higher interfacial surface tension at −0.1 V permits a decrease in the critical size of the EDOT oligomers (or PEDOT nanofibers) needed to become surface active at the L|L interface,^54^ thereby facilitating PEDOT thin film formation.

### Interfacial Electron Transfer in the Presence of Adsorbed EDOT Oligomers and the PEDOT Thin Film at the Aqueous|TFT Interface

The adsorbed EDOT oligomers provide abundant, electrically conductive catalytic sites to catalyze the IET reaction at a much lower overpotential than at a bare L|L interface, as depicted in Figure 2c. Experimentally, a lower overpotential is recorded by the seventh cycle, with the current onset shifting from ca. +0.3 V for the third and fourth cycles (labeled α) to ca. +0.2 V for the seventh cycle (labeled α*) (see Figure 1d). At an applied Δ_o_^w^ϕ ≥ +0.2 V, a huge excess of Ce^4+^ oxidizes the aqueous side of the adsorbed EDOT oligomers, increasing the number of holes within the oligomer chain (decreasing the EDOT oligomers band gap) and thereby decreasing the overpotential needed to oxidize an EDOT monomer on the organic side. Thus, during a CV cycle, the driving force experienced by the adsorbed EDOT oligomers to accelerate IET via this bipolar-like mechanism constantly varies as the interfacial concentration of Ce^4+^ and adsorbed electroactive EDOT oligomers varies, being maximal at an applied Δ_o_^w^ϕ ≥ +0.2 V. The terminology “bipolar-like” is used as a typical bipolar electrode enables electrocatalysis via modulation of their metallic Fermi level, whereas PEDOT has a band gap and does not enable electrocatalysis in such a manner.

EDOT oligomers have a lower oxidation potential than an EDOT monomer.^55^ Thus, once EDOT oligomers are generated in a substantial quantity in the organic side of the EDL, IET may proceed across the full PPW due to their kinetically rapid, autocatalyzed oxidation by adsorbed EDOT oligomers and/or the PEDOT thin film. Consequently, during repetitive CV cycling, a background Faradaic current is always present and contributing to the steady increase in the magnitude of the charge accumulated across the full PPW.

Rapid 2D growth of the PEDOT islands parallel to the L|L interface leads to a PEDOT thin film coating the full interface after ca. 25 cycles at 25 mV·s^–1^, as indicated by the complete disappearance of the sharp negative peak labeled β (Figure 1e). IET then transitions from being purely electrochemically driven to involving simultaneous electrochemical and chemical processes. At this point, EDOT oligomers present in a high concentration in the organic side of the EDL undergo chemical coupling. Likely, EDOT oligomers also chemically oxidize monomers in the diffusion zone of the organic side of the EDL. Simultaneously, the electrochemically polarizable PEDOT thin film continues to oxidize both EDOT monomers and oligomers, as shown schematically in Figure 2d. While many processes can take place, the most dominant reaction is likely the oxidation of EDOT oligomers by the PEDOT thin film. From the 35th cycle onward, the CVs stabilize and only increase very moderately with further cycling (Figure 1e). This may be due to the PEDOT thin film reaching a certain thickness (above 20 nm for example) in excess of that of the ITIES. In this scenario, the entirety of the PEDOT thin film coated L|L interface reaches a similar potential distribution, with the rate of polymerization being controlled by either mass transport (*e.g.,* the diffusion of SO_4_^2–^ counteranions through the film to maintain electroneutrality locally) or the rate of IET. At this stage, it is likely that the PEDOT thin film limits the movement and quantity of ions that charge the interface, like a membrane.

Eventually, when the film reaches a certain thickness by the 40th cycle, significantly exceeding the thickness of the interfacial region directly influenced by the applied , IET continues but the polymerization reaction becomes purely chemical in nature and the four-electrode electrochemical cell can no longer detect the PEDOT thin film’s continued growth, i.e., the CVs remain almost identical with further CV cycling, as shown in Figure 1e for the 40th, 45th, and 50th cycles. Initially, in this final regime, the presence of a high interfacial Ce^4+^ concentration will continue to oxidize the aqueous side of the PEDOT thin film. However, as the interfacial Ce^4+^ concentration is no longer replenished as previously by an applied  positive of the PZC, with time Ce^4+^ is consumed, the kinetics of IET slows considerably (to an almost negligible rate after a certain time), and the potential of the PEDOT thin film falls until only oligomers may be oxidized due to the reduced driving force.

### The Origins of the Capacitive Currents during PEDOT Interfacial Electrosynthesis

**Figure 5:**
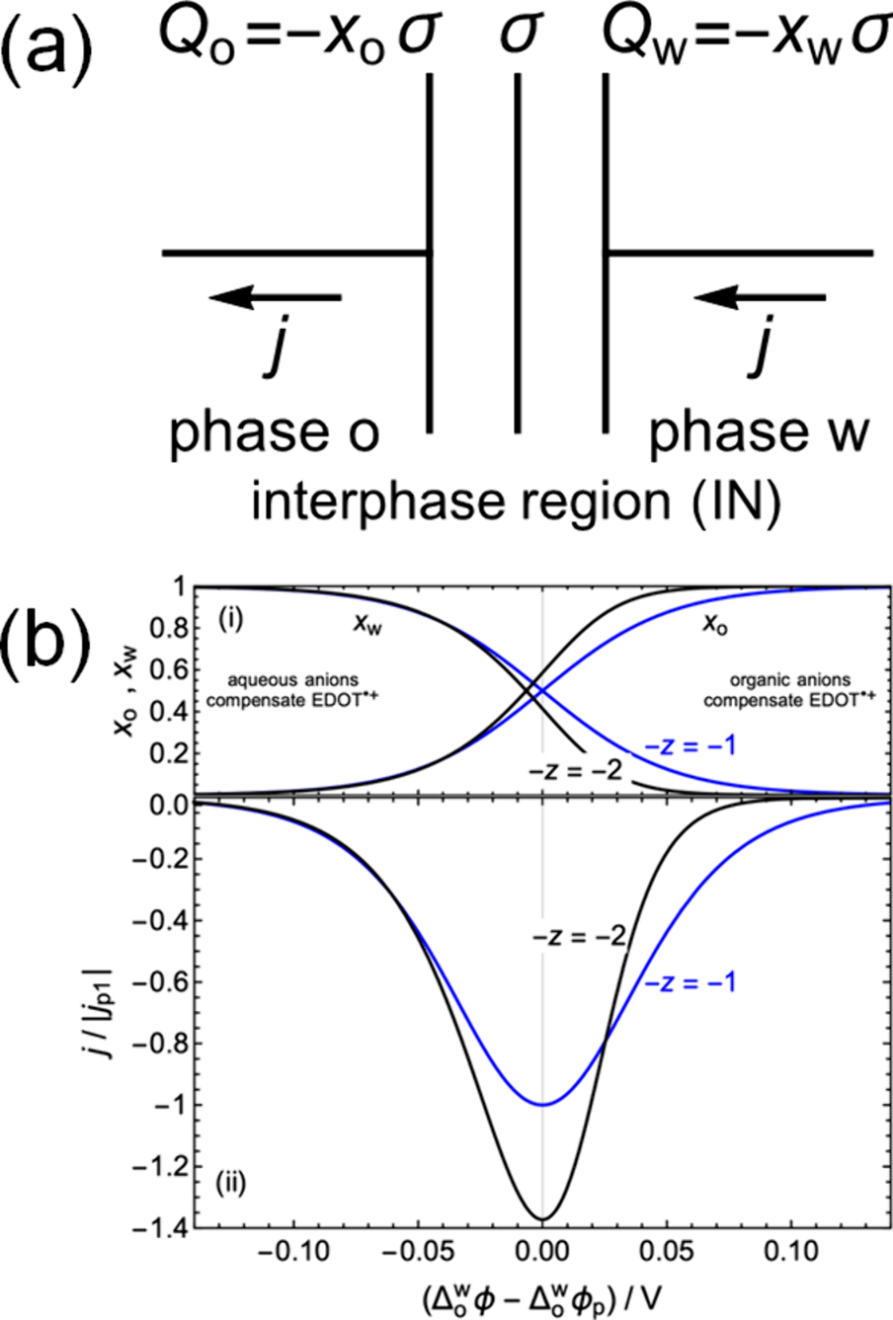
Modeling of peak β as capacitive current due to anion exchange. (a) Schematic representation of the interphase region as a three-plate capacitor. The charge of the cationic EDOT oligomers is compensated by organic and aqueous anions that accumulate in the interphase region. (b) (i) Fraction  of the EDOT charge that is compensated by the organic anions TB^–^ increases with Δ_o_^w^ϕ – Δ_o_^w^ϕ_p_, as described by eq S19. The complementary fraction , compensated by the aqueous anions A^*z*–^, tends to one when Δ_o_^w^ϕ – Δ_o_^w^ϕ_p_ is sufficiently negative. (b) (ii) For monovalent aqueous anions, −*z* = −1, the peak is symmetric (blue curve) (eq S23). For divalent aqueous anions, −*z* = −2, the peak is asymmetric (black curve) (eq S24). Both current densities have been normalized with the peak value *j*_p1_ = *F*νσ/4*RT* for monovalent anions to make clear that the peak area is independent of *z*, where ν is the scan rate.

While numerous processes may lead to capacitive currents that contribute to the charge accumulated as the PEDOT thin film grows (Section S6), capacitive currents due to an anion exchange process as oligomers adsorb at the L|L interface and reversible proton adsorption/desorption to the growing or fully grown PEDOT thin film are the most relevant for PEDOT interfacial electrosynthesis. A point to note is that pseudocapacitance cannot be recorded at the polarizable aqueous|TFT interface as, in a major contrast with PEDOT thin films on solid electrode surfaces, doping/dedoping processes are not taking place during CV cycling at the polarizable L|L interface. The sharp negative peak that occurs at ca. −0.1 V on the backward scan of the CVs during initial cycling (labeled β in Figures 1d,e) is modeled in Section S7 and shown to be purely capacitive in nature. When no charge transfer occurs, the interphase region (IN) that separates the two immiscible aqueous (w) and organic (o) electrolyte solutions behaves similarly to a capacitor with three plates (see Figure 5a). The surface charge density  in a middle plate with no contacts represents the cationic EDOT oligomers in region IN, which suffer no redox process. Their associated charge is described in terms of the concentration  of EDOT monomers with charge number +1. That is, the surface charge density due to EDOT is
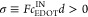
2where *F* is the Faraday constant and *d* is the thickness of region IN. The anions from phases o and w accumulate in region IN. Their respective surface charge densities *Q*_o_ and *Q*_w_ reside in the plates “connected” to phases o and w. The global electroneutrality of region IN requires
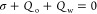
3

It can also be formulated as
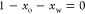
4where the fraction of the charge of the EDOT oligomers that is compensated by the organic (aqueous) anions is *x*_o_ ≡ −*Q*_o_/σ (*x*_w_ ≡ −*Q*_w_/σ).

The distribution of the anions in the interphase region IN is modulated by the applied Δ_o_^w^ϕ. The fraction *x*_o_ is also determined by *K*_TB_ and *K*_A_, the chemical partition coefficients of the organic anions TB^–^ and aqueous anions A^*z*–^, respectively. In turn, *K*_TB_ and *K*_A_ determine the peak potential Δ_o_^w^ϕ_p_. In other words, the fraction  of the charge of the cationic EDOT oligomers that is compensated by the organic anions TB^–^ tends to one with increasing Δ_o_^w^ϕ – Δ_o_^w^ϕ_p_, as described by eq S19 and illustrated in Figure 5b(i). The complementary fraction , compensated by the aqueous anions A^*z*–^, tends to one when Δ_o_^w^ϕ – Δ_o_^w^ϕ_p_ is sufficiently negative (see Figure 5b(i)). A key prediction of the model in Section S7 is that for monovalent aqueous anions, −*z* = −1, the peak shape is symmetric (blue curve), as described by eq S23 and illustrated in Figure 5b(ii). Meanwhile, for divalent aqueous anions, −*z* = −2, the peak shape is asymmetric (black curve) (see eq S24 and Figure 5b(ii)). The shape of the latter peak is in very good agreement with the shape of the peak β experimentally observed when using sulfuric acid as the aqueous electrolyte (see Figure 1d).

A recovered PEDOT thin film was reimmobilized at a polarizable aqueous|TFT interface without the aqueous Ce^4+^ oxidant or organic EDOT monomer present. CV was carried out varying the aqueous phase pH using the electrochemical cell configuration described in Figure S8a. At ca. pH 0.7 using a 0.2 M H_2_SO_4_ aqueous electrolyte, significant peaks showing sharp increases in current indicative of surface or adsorption processes at the polarizable L|L interface were recorded on the forward and reverse scans (Figure S8b). These peaks correspond to those labeled γ and γ* in Figure 1e that were seen to dominate the voltammetric profile by the 25th cycle during PEDOT interfacial electrosynthesis. The intensity of the peaks is pH dependent, with a large decrease in magnitude observed when the aqueous phase was phosphate-buffered saline (PBS) solution (ca. pH 7), and their disappearance entirely with an aqueous solution of 0.01 M NaOH (ca. pH 10). Thus, given this pH dependence, the peaks are attributed to reversible proton adsorption/desorption on the growing PEDOT thin film.

The PEDOT thin film reimmobilized at the polarizable L|L interface is in the interphase region IN. The film has a molar concentration  of EDOT monomers with charge number +1, and a concentration *c*_site_^IN^ of sites for reversible adsorption of protons. Given that the PEDOT film is likely to be formed by chains of relatively low molar mass, *c*_site_^IN^ is likely to be high. The surface charge density  electrostatically hinders the adsorption of protons. However, the adsorption can occur due to a strong “chemical affinity”.^56^ He et al. have shown using density functional theory calculations that octa-EDOT adduction with protons on the carbon of the end-thiophene releases a huge energy of 1.15 MJ·mol^–1^ (with respect to the neutral octa-EDOT).^56^ Thus, the experimentally observed signal was modeled in Section S8, with the proton adsorption peak calculated using eq S28 exhibiting an asymmetric shape (Figure S8c) that matches very well with the experimental one (Figure S8b, 0.2 M H_2_SO_4_).

### Scan Rate Studies of Potentiodynamic PEDOT Interfacial Electrosynthesis

**Figure 6:**
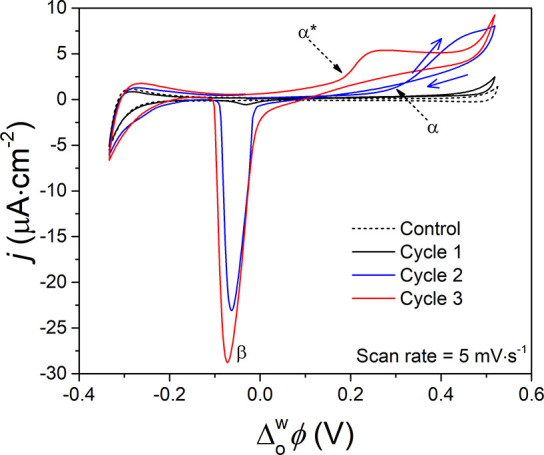
Observation of a “nucleation loop” during interfacial electrosynthesis. CVs were recorded using the electrochemical cell configuration described in Figure 1a. For control experiments, the Ce(SO_4_)_2_ concentration was 0 mM (dashed black CV). A nucleation loop is observed at positive applied Δ_o_^w^ϕ during the second CV cycle (blue CV). CVs were recorded under ambient, aerobic conditions at a scan rate of 5 mV·s^–1^.

The first three CV cycles of PEDOT interfacial electrosynthesis, using the electrochemical cell configuration described in Figure 1a, at a scan rate of 5 mV·s^–1^ are shown in Figure 6. As the scan direction is reversed in cycle 2 at the positive edge of the PPW, a so-called nucleation loop is recorded. Such CV trace crossings are common in conventional electropolymerization at solid electrode|electrolyte interfaces and associated with a homogeneous reaction between oligomer intermediates and the initial monomer.^57^ Thus, the “nucleation loop” in this instance is somewhat of a misnomer, not being a result of nucleation processes during electropolymerization but actually due to homogeneous reactions.

EDOT^*n*+^ oligomers are rapidly generated in the organic diffuse layer at positive applied  during the forward scan in Figure 6. These oligomers may undergo a homogeneous comproportionation reaction in the organic diffuse layer with neutral EDOT monomers and become partially reduced to EDOT^(*n*–1)+^. Then, upon reversing the cycle direction, the EDOT^(*n*–1)+^ species are reoxidized to EDOT^*n*+^ by IET with Ce^4+^ (leading to the CV trace crossover). In line with the descriptions by Heinze et al. of electropolymerization at solid electrode|electrolyte interfaces,^57^ this interpretation of a nucleation loop prerequisites that (i) the standard redox potentials of the redox active oligomer (EDOT^(*n*–1)+^) and EDOT monomer are close, with that of the monomer being more positive than that of the oligomer, and (ii) the rate of the IET reaction between Ce^4+^ and the EDOT monomer at the bare L|L interface must be sluggish. The latter slows the depletion of neutral EDOT in the organic diffuse layer, thereby enabling the homogeneous oxidation of EDOT via a comproportionation reaction to proceed (or compete). This autocatalytic mechanism thereby considerably facilitates the starting oxidation of neutral EDOT,^57^ with dramatic changes in the CVs (associated with oligomer adsorption and PEDOT thin film formation) observed in the cycles immediately following the cycle containing the nucleation loop.

To further probe the influence of scan rate on PEDOT thin film electrosynthesis, CV cycling experiments were carried out at 1 and 100 mV·s^–1^, as described in Section S9. Using aqueous Ce^4+^ concentrations of either 2 or 4 mM (Figures S9 and S10), the PEDOT thin films formed at 100 mV·s^–1^ were notably thinner and had a more metallic or “golden” color (Figure S11) than a PEDOT thin film formed at 25 mV·s^–1^ under otherwise identical conditions. A high concentration of EDOT oligomers is confined to a thinner layer in the organic side of the EDL at 100 mV·s^–1^ than at 25 mV·s^–1^, potentially leading to differences in the morphology and thickness of the film formed. At the lower scan rate of 1 mV·s^–1^, no PEDOT interfacial film was formed after cycling using aqueous Ce^4+^ concentrations of either 2 or 6 mM, respectively (Figure S12). This was despite the characteristic CVs of PEDOT interfacial electrosynthesis being observed during the initial cycles with 6 mM Ce^4+^ (Figures S12b and S13). At 1 mV·s^–1^, the relatively long contact time with the large excess of interfacial Ce^4+^ may deplete the interfacial concentration of EDOT monomers and overoxidize oligomers present in the organic diffuse layer. These overoxidized oligomers may then be incapable of forming longer chains during subsequent CV cycles to reach the critical chain length. Direct visual evidence of such overoxidation was the coating of the liquid|liquid interface with a white precipitate after cycling at 1 mV·s^–1^ (Figure S14). The latter immediately redissolved upon agitating the L|L electrochemical cell. The lack of PEDOT thin film formation at a slow scan rate is supported by observations from chemical oxidative polymerization of CPs such as poly(aniline) and poly(pyrrole) where the CP yield is dependent on the amount of oxidant until the monomer is completely consumed and then starts to decrease at oxidant:monomer ratios in excess of just 1.25:1 owing to oligomer overoxidation.^58^

## Conclusions

In this article, for the first time, electrochemical responses typically observed for CP electropolymerization by repetitive CV cycling at a solid electrode|electrolyte interface are clearly replicated for CP electrosynthesis at a polarizable L|L interface. Most prominently, a steady buildup of charge occurs with successive cycles and a nucleation loop appears during an early cycle. The CVs recorded during interfacial electrosynthesis continually evolve with cycling due to the changing nature of the Faradaic and capacitive processes taking place at the L|L interface as EDOT oligomers adsorb and the PEDOT thin film grows. To prevent overoxidation during interfacial electrosynthesis, faster scan rates of 25 or 100 mV·s^–1^ were optimal, with slower scan rates of 1 or 5 mV·s^–1^ failing to yield PEDOT thin films during cycling. Through (spectro)electrochemical experiments and modeling, a comprehensive understanding of the evolving features of the CVs during cycling has been achieved. This thorough understanding of the mechanistic origins of each feature of the evolving CVs opens the prospect of probing each step during interfacial electrosynthesis of any suitable oxidant/monomer combination that yields a CP thin film with exceptional detail. In turn, this will lead to opportunities to develop feedback mechanisms to tune CP thin film structure–activity relationships, whereby the effects of any experimental variable on one particular aspect of interfacial electrosynthesis, for example the IET or oligomer adsorption steps, may be monitored electrochemically and related to changes of the physical properties (morphology, crystallinity, *etc.*) and in turn activities (conductivity, catalytic activity, *etc.*) of the CP thin films produced.

## Data Availability

The Mathematica notebook file used to generate Figures 3a and 5b and Figure S8c are available at Zenodo (10.5281/zenodo.12206217).

## Supporting Information Available

Supporting Information and methods; electrochemistry of preformed EDOT oligomers at a polarizable aqueous|TFT interface; discussion on the onset interfacial Galvani potential difference for IET of a biphasic single-step IET reaction; modeling the effect of the applied  on the ionic distributions in the interfacial region; spectroelectrochemical UV–vis-NIR analysis of a PEDOT thin film; discussion on capacitive currents contributing to the steady growth of the charge accumulated during interfacial electrosynthesis; modeling of peak β as capacitive current due to anion exchange; modeling the capacitive current peak due to proton adsorption; scan rate studies of PEDOT interfacial electrosynthesis (PDF)
