# Supplementary material for: Mechanistic Insights into the Potentiodynamic Electrosynthesis of PEDOT Thin Films at a Polarizable Liquid|Liquid Interface
Source: J Am Chem Soc. 2024 Oct 9;146(42):28941–51. doi: 10.1021/jacs.4c09638 (PMC11505374; doi:10.1021/jacs.4c09638)
Supplement: Supplementary file 1 — ja4c09638_si_001.pdf [file ja4c09638_si_001.pdf]

Supporting Information

Mechanistic Insights into the Potentiodynamic Electrosynthesis of PEDOT Thin Films at a Polarisable Liquid|Liquid Interface

Rob A. Lehane,[a] Alonso Gamero-Quijano,*[a,b] José A. Manzanares,[c] Micheál D. Scanlon*[,a]

[a] The Bernal Institute and Department of Chemical Sciences, School of Natural Sciences, University of Limerick (UL), Limerick V94 T9PX, Ireland.

[b] Instituto de Catálisis y Petroleoquímica − Consejo Superior de Investigaciones Científicas (ICP − CSIC), Calle de Marie Curie 2, Madrid 28049, Spain.

[c] Department of Thermodynamics, Faculty of Physics, University of Valencia, c/Dr. Moliner, 50, Burjasot E-46100 Valencia, Spain.

*E-mail: alonso.gamero@icp.csic.es; micheal.scanlon@ul.ie

Table of Contents

Section	Contents	Page
**S1**	Supplementary materials and methods	3
**S2**	Electrochemistry of pre-formed EDOT oligomers at a polarisable aqueous|TFT interface	7
**S3**	The onset interfacial Galvani potential difference for IET ($\Delta_o^w \phi_{IET}^{onset}$) of a biphasic single-step IET reaction	10
**S4**	Effect of the applied $\Delta_o^w \phi$ on the ionic distributions in the interfacial region	12
**S5**	Spectroelectrochemical UV-vis-NIR analysis of a PEDOT thin film	21
**S6**	Capacitive currents contributing to the steady growth of the charge accumulated during interfacial electrosynthesis	24
**S7**	Modelling of peak β as capacitive current due to anion exchange	25
**S8**	Modelling the capacitive current peak due to proton adsorption	29
**S9**	Scan rate studies of PEDOT interfacial electrosynthesis	32
	Supplementary references	40

Section S1. Supplementary materials and methods

Materials. All chemicals were used as received without further purification. All aqueous solutions were prepared with ultra-pure water (Millipore Milli-Q, specific resistivity 18.2 MΩ·cm). The organic solvent α,α,α-trifluorotoluene (TFT, ≥99%) was received from Acros Organics. The organic monomer 3,4-ethylenedioxythiophene (EDOT, 97%), cerium(IV) sulfate ($Ce(SO_4)_2$, 99%), sulfuric acid (H_2SO_4, 95.0%), lithium chloride (LiCl, ≥99%) and tetraethylammonium chloride (TEACl, 99%) were purchased from Sigma-Aldrich. Lithium tetrakis(pentafluorophenyl)borate diethyletherate (LiTB) was received from Boulder Scientific Company. The organic electrolyte bis(triphenylphosphoranylidene)ammonium tetrakis(pentafluorophenyl)borate (BATB) was prepared by metathesis of equimolar solutions of BACl and LiTB in a methanol-water (2:1 v/v) mixture. The resulting precipitates were filtered, washed and recrystallised from acetone.

Electrochemical measurements. All experiments were performed using a custom-made 4-electrode electrochemical cell which is illustrated in Figure S1a. Two reference electrodes positioned on different sides of the L|L interface measured the interfacial Galvani potential difference $\Delta_o^w \phi$. Platinum counter electrodes placed in each phase allowed the flow of electric current. The geometrical surface area of the liquid|liquid (L|L) interface was ~1.66 cm^2. The electrochemical cell was custom made by Mulholland Scientific Glassblowing (Southampton, UK). The aqueous reference electrode was a commercial LowProfile Ag/AgCl KCl gel electrode from Pine research (USA). The organic reference electrode was a Ag/AgCl wire immersed in an organic reference solution (10 mM LiCl and 1 mM BACl in ultra-pure water) that established a liquid junction with the organic phase.

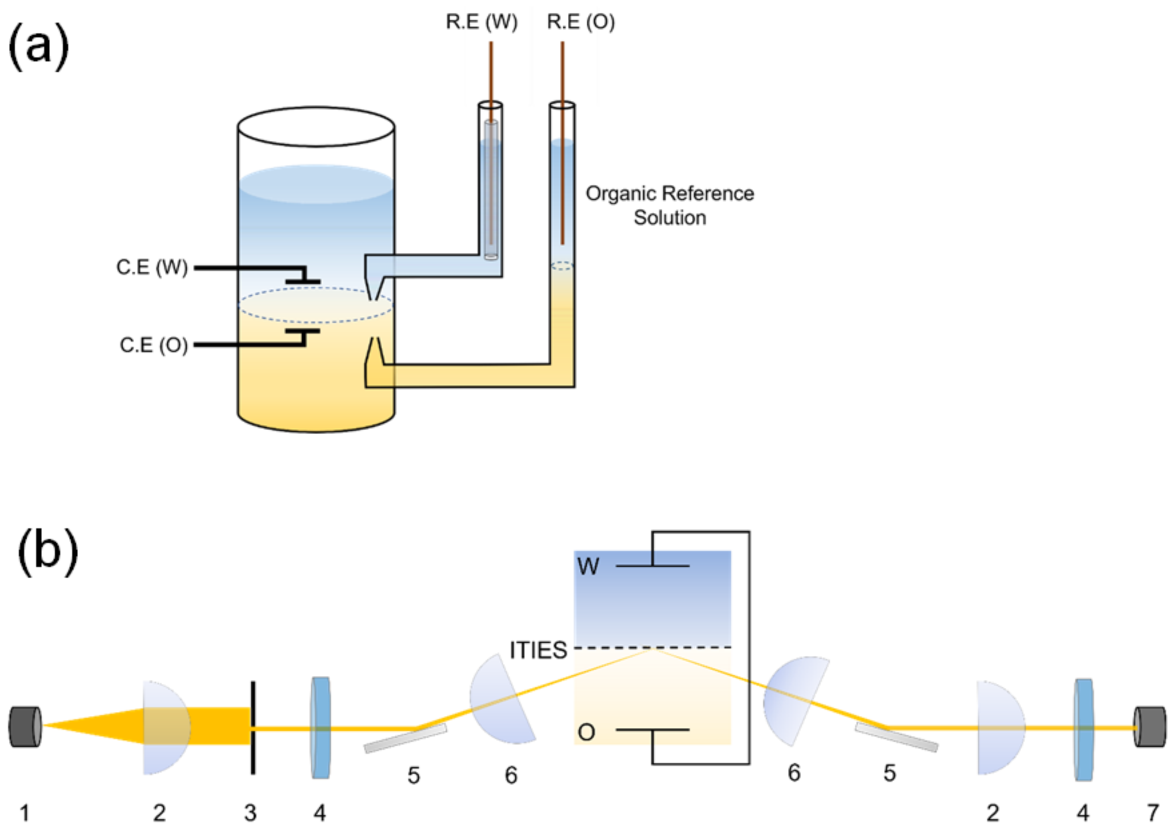

Figure S1. (a) Schematic of the 4-electrode electrochemical cell to carry out potentiodynamic electrosynthesis at an interface between two immiscible electrolyte solutions. Platinum counter electrodes (C.E) were placed in each phase to allow the flow of electric current. Reference electrodes (R.E) centred above and below the liquid|liquid (L|L) interface, were used to the measure the interfacial Galvani potential difference $\Delta_o^w\phi$. The aqueous R.E was a Ag/AgCl wire in a concentrated KCl gel. The organic R.E was a Ag/AgCl wire immersed in the organic reference solution. The geometrical area of the L|L interface was ~1.66 cm^2. **(b)** The total internal reflection (TIR) configuration used to obtain *in situ* UV/vis absorption spectra measurements. (1) Xe light source (Ocean Optics) (2) UVFS coated plano convex lenses (Thorlabs), (3) iris diaphragm (Thorlabs), (4) neutral density (ND) filter (Thorlabs), (5) 'Hot' mirror (cut-off wavelength 800 nm) (Thorlabs), (6) Achromatic doublets (Thorlabs), and (7) Maya2000 Pro spectrometer.

The applied $\Delta_o^w\phi$ was controlled externally using an Autolab PGSTAT204 from Metrohm (Netherlands), in conjunction with NOVA software version 2.1.2. Differential capacitance was calculated from the interfacial admittance recorded using an Autolab FRA32M module in combination with the Autolab PGSTAT204 at a frequency of 10 Hz and root mean square (RMS) amplitude of 10 mV. All electrochemical measurements were carried out in ambient aerobic conditions at room temperature (RT, 21°C). Measurements were calibrated to the Galvani potential scale using the following the relationship $E = \Delta_o^w\phi + \Delta E_{ref}$. The critical value of ΔE_{ref} was determined using the electrochemical half-wave ion transfer response of tetraethylammonium cations (TEA^+; $E_{1/2,TEA^+}^{w\rightarrow TFT}$) and the standard ion transfer potential of TEA^+ from the aqueous to TFT phase (known to be $\Delta_o^w\phi_{tr,TEA^+}^{\ominus,w\rightarrow TFT} = 0.149$ V).[1]

Cleaning procedure for PEDOT thin films extracted from the L|L interface. The PEDOT thin film formed at the aqueous|TFT interface after interfacial electrosynthesis is flexible and robust. Prior to any *ex situ* characterisation, the film may be carefully scooped from the L|L interface and transferred from one glass vial to another in series, each containing a cleaning solution. The first vial contains 20 mL of HPLC grade acetone. EDOT monomers are highly soluble in acetone, with a solubility above 133.1 $g{\cdot}L^{-1}$ (determined in this work) compared to water, where the solubility is 2.1 $g{\cdot}L^{-1}$.[2] In addition to removing any remaining EDOT monomers from the PEDOT thin film, cleaning in acetone also removes any remnants of the organic BATB electrolyte salt. The second and third cleaning solutions contain 20 mL of 80:20 and 50:50 (v/v) 0.2 M H_2SO_4: acetone mixtures, respectively. In these mixtures, the H_2SO_4 removes remnants of the $Ce(SO_4)_2$ salt while also inhibiting spontaneous de-doping of the PEDOT thin films due to a Donnan potential created at the interface of the polymer film. This cleaning procedure was repeated in triplicate for each PEDOT thin film analysed by any *ex situ* method.

Spectroscopic measurements. *In situ* UV/vis absorption spectra in total internal reflection mode (TIR-UV/vis) were recorded at the polarisable L|L interface using the setup described in Figure 1b and experimental methodology described previously by Molina-Osorio *et al.*[3] In this optical setup, the light source passes through the organic side of the electrical double layer (EDL) and is reflected at the L|L interface at an obtuse angle. As a result, the TIR-UV/vis absorbance spectra may contain signals from species either in the organic side of the EDL at the L|L interface, adsorbed on the L|L interface, or a combination of both. A silanised 4-electrode electrochemical cell was used to ensure that L|L interface was entirely flat. To ensure TIR conditions, lenses, a diaphragm, and mirrors were used to focus the light source onto the L|L interface at an angle of incidence of 75°. The latter is greater than the critical angle at an aqueous|TFT interface, determined to be ca. 70° previously.[3] With lenses, the reflected light was focused onto an optical fibre connected to the spectrometer. Neutral density filters were used to reduce the intensity of the light. The heating of the interfacial region was reduced with hot mirrors that cut wavelengths above 700 nm. Under TIR conditions, the penetration depth of the evanescent field on the aqueous side of the L|L interface is ca. 200 nm.[4] The L|L interface was established 30 min prior to the start of the *in situ* measurements to decrease any baseline drift during the TIR-UV/vis measurements. Furthermore, before the addition of 1 mL of oxidant to the aqueous phase, that same volume was removed from the blank aqueous electrolyte solution (used to obtain the "blank" spectra), so that the position of the L|L interface remained constant. Measurements were made with a Maya 2000 Pro Spectrometer (Ocean Optics, USA) and Xe light source (Ocean Optics, USA). The spectrometer was controlled with OceanView software, version 1.6.7.

Section S2. Electrochemistry of pre-formed EDOT oligomers at a polarisable aqueous|TFT interface

To examine the nature of the EDOT oligomers in the organic solution of 5 mM BATB in TFT, pre-formed EDOT oligomers were added to the electrochemical cell configuration described in Scheme S1. The EDOT oligomers were prepared potentiostatically using a 3-electrode configuration with a platinum polycrystalline electrode working electrode in a stirred solution of 5 mM EDOT and 5 mM BATB in TFT solvent. The counter electrode was a Pt wire. The reference electrode was a Ag/AgCl wire, which was immersed in an "organic reference solution" that established a liquid junction with the organic solvent. The organic reference solution was an aqueous solution of 1 mM LiCl and 10 mM BACl. The measured potential was calibrated to the standard hydrogen electrode (SHE) potential scale using the decamethyferrocene ($DcMFc^+$/DcMFc) redox couple in TFT, which has a standard redox potential of +0.107 V (*vs.* SHE).[5] For the electrosynthesis, a potential of 1.44 V (*vs.* SHE) was applied for 30 min. After this time, the organic solution appeared a translucent blue colour which signified the presence of EDOT oligomers. This EDOT oligomer solution was then filtered with an organic solvent compatible syringe filter to remove any insoluble PEDOT particles. Finally, an aliquot of this solution (20 μL) was added directly to the organic phase of the 4-electrode electrochemical cell (with no monomer or oxidant present as described in Scheme S1). For this experiment, the 4-electrode electrochemical cell had an extra capillary into the organic phase to allow the addition of reactants after the L|L interface was formed. Furthermore, the organic phase was stirred with a stirring bar to improve the dispersion of the EDOT oligomers after their addition.

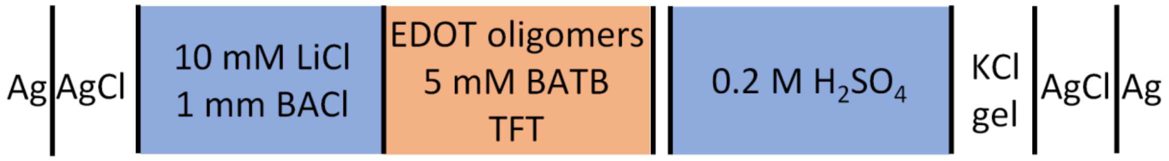

Scheme S1. Electrochemical cell configuration of the four-electrode electrochemical cell employed to investigate the electrochemistry of EDOT oligomers at a polarisable aqueous|TFT interface. The EDOT oligomer solution was prepared potentiostatically using a 3-electrode electrochemical setup with a platinum polycrystalline electrode in a solution of 5 mM EDOT and 5 mM BATB in TFT solvent.

The electrochemical response before and after the addition of EDOT oligomers to the organic phase is shown in Figure S2. The dashed line shows the blank voltammogram in the absence of EDOT oligomers. On the forward scan, the increase of positive current seen at +0.4 V corresponds to the transfer of protons from the aqueous to the organic phase. On the reverse scan, the increase of negative current at –0.3 V corresponds to the transfer of SO_4^{2-} anions to the organic phase. The solid lines show the CVs after the addition of the EDOT oligomer solution. The main feature is a pair of reversible peaks centred at ca. 0 V, which increase in magnitude on subsequent CV cycles. This signal can be attributed to the presence of cationic EDOT oligomer species in the organic phase that have a half-wave ion transfer potential of ca. 0 V. A second set of reversible peaks can be seen at ca. +0.3 V, which could be due to transfer of larger EDOT oligomers. Furthermore, a steady growth of the charge accumulated with each successive cyclic voltammetry (CV) cycle in the EDL indicates possible EDOT oligomer assembly at the L|L interface. Based on these observations, the EDOT oligomers generated in the organic phase during interfacial electrosynthesis are clearly ionic in nature and logically positively charged.

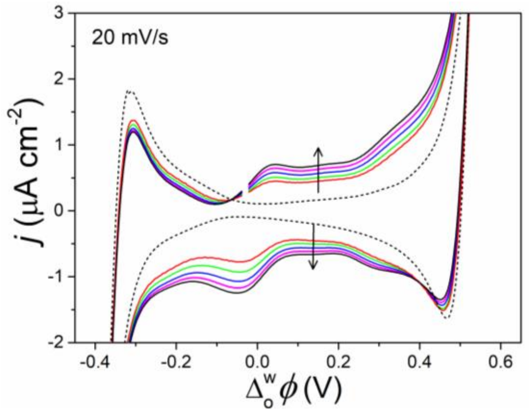

Figure S2. Cyclic voltammograms (CVs) with pre-formed EDOT oligomers present in the organic phase were recorded using the electrochemical cell configuration described in Scheme S1. A control voltammogram (dashed line) was obtained prior to the addition of EDOT oligomers. The scan rate used was 20 mV·s^{-1}.

Section S3. The onset interfacial Galvani potential difference for IET ($\Delta_o^w \phi_{IET}^{onset}$) of a biphasic single-step IET reaction

During PEDOT interfacial electrosynthesis, electrons flow from the organic to aqueous phase and thus, by convention, a positive current should be measured.[6] These positive Faradaic currents (labelled α) appear at a positive applied $\Delta_o^w \phi$, with an onset interfacial Galvani potential difference for IET ($\Delta_o^w \phi_{IET}^{onset}$) approaching +0.3 V, see Figure 1d for the 3rd cycle at 25 mV·s^{-1}. The standard Galvani potential difference for IET is defined as

$$\Delta_o^w \phi_{IET}^{\ominus} = \left[E_{EDOT^{\bullet +}/EDOT}^{\ominus}\right]_{SHE}^{o} - \left[E_{Ce^{4+}/Ce^{3+}}^{\ominus}\right]_{SHE}^{w} . \quad (S1)$$

Previously, we have determined $\Delta_o^w \phi_{IET}^{\ominus}$ as ca. 0 V by CV using 3-electrode electrochemical cells to separately determine (i) the redox potential of the aqueous $Ce^{4+/3+}$ oxidant redox couple in 0.2 M H_2SO_4 and (ii) the onset potential of EDOT oxidation in a solution of TFT containing BATB electrolyte, both *versus* the standard hydrogen electrode.[7] We confirmed $\Delta_o^w \phi_{IET}^{\ominus}$ as ca. 0 V with the same aqueous and organic solutions by CV through a direct measurement using a closed bipolar electrochemical cell methodology.[7] As $\Delta_o^w \phi_{IET}^{\ominus}$ is assumed to be equal to $\Delta_o^w \phi_{1/2}$ for a reversible biphasic single-step IET reaction, then IET from the EDOT monomers to Ce^{4+} is not predicted to be thermodynamically spontaneous. For spontaneity, $\Delta_o^w G_{IET}^{\ominus}$ must be negative according to

$$\Delta_o^w G_{IET}^{\ominus} = nF\Delta_o^w \phi_{IET}^{\ominus} , \quad (S2)$$

where n is the number of electrons transferred during the biphasic single-step IET reaction and $\Delta_o^w G_{IET}^{\ominus}$ is the standard Gibbs energy of IET when the direction of electron flow is from an organic to aqueous redox couple. To drive interfacial electrosynthesis, external application of a positive $\Delta_o^w \phi$ provides a thermodynamic driving force to accelerate IET from the EDOT monomers to Ce^{4+} since

$$RT \ln \frac{c_{\mathrm{EDOT}^{\bullet+}}\, c_{\mathrm{Ce}^{3+}}}{c_{\mathrm{EDOT}}\, c_{\mathrm{Ce}^{4+}}} \approx F(\Delta_{\mathrm{o}}^{\mathrm{w}}\phi - \Delta_{\mathrm{o}}^{\mathrm{w}}\phi_{\mathrm{IET}}^{\ominus}) \ . \tag{S3}$$

Recently we demonstrated that $\Delta_{\mathrm{o}}^{\mathrm{w}}\phi_{\mathrm{IET}}^{\mathrm{onset}}$ of a biphasic single-step IET reaction does not necessarily correlate with the thermodynamically determined $\Delta_{\mathrm{o}}^{\mathrm{w}}\phi_{\mathrm{IET}}^{\ominus}$.[8] The latter is true even if $\Delta_{\mathrm{o}}^{\mathrm{w}}\phi_{\mathrm{IET}}^{\ominus}$ is negative on the Galvani scale and IET between the two redox couples is therefore predicted to be thermodynamically spontaneous according to Equation S2 in the absence of any externally applied driving force. Instead, $\Delta_{\mathrm{o}}^{\mathrm{w}}\phi_{\mathrm{IET}}^{\ominus}$ in Equation S3 had to be replaced by the potential of zero charge at the polarisable L|L interface, $\mathrm{PZC} = \Delta_{\mathrm{o}}^{\mathrm{w}}\phi_{\mathrm{IET}}^{\ominus} + \eta_{\mathrm{PZC}}$, where η_{PZC} is the required overpotential (*i.e.,* applied $\Delta_{\mathrm{o}}^{\mathrm{w}}\phi$ beyond the value of $\Delta_{\mathrm{o}}^{\mathrm{w}}\phi_{\mathrm{IET}}^{\ominus}$) to reach the PZC. Thus, $\Delta_{\mathrm{o}}^{\mathrm{w}}\phi_{\mathrm{IET}}^{\mathrm{onset}}$ was shown to be highly dependent on the kinetics of the IET reaction, which are largely dictated by the potential-modulated interfacial concentration of each redox species. The latter, in turn, is dependent on $\Delta_{\mathrm{o}}^{\mathrm{w}}\phi - \mathrm{PZC}$ and, therefore, $\Delta_{\mathrm{o}}^{\mathrm{w}}\phi_{\mathrm{IET}}^{\mathrm{onset}}$ can be defined in terms of the PZC and η_{polarise} as described in Equation 1 in the main article.

Section S4. Effect of the applied $\Delta_o^w\phi$ on the ionic distributions in the interfacial region

For the electrochemical cell configuration outlined in Figure 1a in the absence of EDOT monomers, the effect of the applied $\Delta_o^w\phi = \phi^w - \phi^o$ on the distribution of ionic species (Ce^{4+}, H^+, SO_4^{2-}, BA^+, and TB^-) in the interfacial region has been determined by solving the Poisson-Boltzmann equation (PBE). In phase o, the steric effect has been taken into account by using the Bikerman equation.[9–11] That is, since the organic ions BA^+ and TB^- are *bulky*, it is assumed that their concentration cannot exceed $c_{o,max}$. A value $c_{o,max}$ = 2 M has been used as a reasonable guess; the implications of the value of $c_{o,max}$ are discussed below in this section.

The planar interface, at $x = 0$, is assumed to be ideally polarisable, with no ion or electron transfer processes taking place. The PZC is arbitrarily set at 0 V. The bulk aqueous phase (phase w) at $x > 0$ has $c_{b,w}$ = 200 mM of H_2SO_4 and $c_{b,Ce}$ = 2 mM of $Ce(SO_4)_2$. Although at 200 mM, 69 % of sulfuric acid is dissociated as bisulfate ions and 31 % as sulfate ions, for the sake of simplicity we assume there are no bisulfate ions. The bulk organic phase (phase o) at $x < 0$ has $c_{b,o}$ = 5 mM of BATB.

When $\Delta_o^w\phi$ is applied to this ideally polarisable interfacial system, charge is accumulated around the interface $x = 0$. The charge density Q_w in the aqueous side of the EDL is equal in magnitude and opposite in sign to the charge density Q_o in the organic side,

$$Q_w = Q_{H^+} + Q_{Ce^{4+}} + Q_{SO_4^{2-}} = -Q_{BA^+} - Q_{TB^-} = -Q_o \ , \qquad \text{(S4)}$$

where the contribution of ionic species i is $Q_i = z_i F \int_{EDL} [c_i(x) - c_{b,i}] dx$, and the integral extends over the (aqueous or organic side of the) EDL where species i resides. The equilibrium condition requires that the electrochemical potential of species i is independent of position. Its

concentration $c_i(x)$ is then related to the local electric potential $\phi(x)$ through the Bikerman equation[9–11]

$$\frac{c_i(x)}{1-\sum_i v_i c_i(x)} \mathrm{e}^{z_i f \phi(x)} = \frac{c_{\mathrm{b},i}}{1-\sum_i v_i c_{\mathrm{b},i}} \mathrm{e}^{z_i f \phi_{\mathrm{b},i}} , \quad \text{(S5)}$$

where v_i and z_i are the (partial) molar volume and charge number of ionic species i, and $f = F/RT$. The potential $\phi_{\mathrm{b},i}$ is ϕ^{o} for the ions in phase o and ϕ^{w} for those in phase w. Introducing the dimensionless potential $\varphi(x) \equiv f[\phi(x) - \phi^{\mathrm{o}}]$, and denoting the similar (partial) molar volumes of BA^+ and TB^- ions as $1/c_{\mathrm{o,max}}$, the Bikerman equation simplifies to

$$c_{\mathrm{BA}^+}(x)\mathrm{e}^{\varphi(x)} = c_{\mathrm{TB}^-}(x)\mathrm{e}^{-\varphi(x)} = \frac{c_{\mathrm{b,o}}}{1+\phi_{\mathrm{o,steric}}[\cosh\varphi(x)-1]} , \ (x < 0) \quad \text{(S6)}$$

where $\phi_{\mathrm{o,steric}} \equiv 2c_{\mathrm{b,o}}/c_{\mathrm{o,max}}$ is known as the steric fraction. Note that $\varphi(x) \gg 1$ implies a local accumulation of TB^- ions such that their volume fraction in the solution is practically equal to one, $c_{\mathrm{TB}^-}(x) \approx c_{\mathrm{o,max}}$; and, similarly, $-\varphi(x) \gg 1$ implies $c_{\mathrm{BA}^+}(x) \approx c_{\mathrm{o,max}}$. When the steric fraction is sufficiently small, the Bikerman equation reduces to the Boltzmann equation. This approximation is valid for the aqueous ions, whose equilibrium distributions are given by

$$c_{\mathrm{H}^+}(x) = 2c_{\mathrm{b,w}}\mathrm{e}^{-[\varphi(x)-f\Delta_{\mathrm{o}}^{\mathrm{w}}\phi]} , \ c_{\mathrm{Ce}^{4+}}(x) = c_{\mathrm{b,Ce}}\mathrm{e}^{-4[\varphi(x)-f\Delta_{\mathrm{o}}^{\mathrm{w}}\phi]} ,$$

$$c_{\mathrm{SO}_4^{2-}}(x) = (c_{\mathrm{b,w}} + 2c_{\mathrm{b,Ce}})\mathrm{e}^{2[\varphi(x)-f\Delta_{\mathrm{o}}^{\mathrm{w}}\phi]} , \ (x > 0) . \quad \text{(S7)}$$

In the organic side of the EDL ($x < 0$), the Poisson equation is

$$\frac{\mathrm{d}^2\varphi}{\mathrm{d}x^2} = -\frac{fF}{\epsilon_0\epsilon_{\mathrm{r,o}}}(c_{\mathrm{BA}^+} - c_{\mathrm{TB}^-}) = \kappa_o^2 \frac{\sinh\varphi}{1+\phi_{\mathrm{o,steric}}(\cosh\varphi - 1)} , \quad \text{(S8)}$$

where $\kappa_{\mathrm{o}}^2 \equiv 2F^2 c_{\mathrm{b,o}}/\epsilon_0\epsilon_{\mathrm{r,o}}RT$ is the (squared) Debye parameter in phase o, ϵ_0 is the vacuum permittivity and $\epsilon_{\mathrm{r,o}}$ is the relative permittivity of phase o. The integration of Equation S8 between the bulk organic phase (where $\varphi = 0$ and $\mathrm{d}\varphi/dx = 0$) and $x < 0$ gives

$$\left(\frac{\mathrm{d}\varphi}{\mathrm{d}x}\right)^2 = \frac{2\kappa_o^2}{\phi_{\mathrm{o,steric}}}\ln\{1 + \phi_{\mathrm{o,steric}}[\cosh\varphi(x) - 1]\}\,. \tag{S9}$$

The integral of the space charge density over the EDL is the surface charge density in the EDL. Thus, Equation S9 implies that the surface charge density $Q_\mathrm{o} = Q_{\mathrm{BA}^+} + Q_{\mathrm{TB}^-} = F\int_{-\infty}^{0}[c_{\mathrm{BA}^+}(x) - c_{\mathrm{TB}^-}]\mathrm{d}x = -(\epsilon_0\epsilon_{\mathrm{r,o}}/f)\mathrm{d}\varphi/\mathrm{d}x|_{x\to 0-}$ satisfies

$$\frac{Q_\mathrm{o}^2}{2\epsilon_0 RT} = \frac{2\epsilon_{\mathrm{r,o}}c_{\mathrm{b,o}}}{\phi_{\mathrm{o,steric}}}\ln\{1 + \phi_{\mathrm{o,steric}}[\cosh\varphi(0) - 1]\}\,. \tag{S10}$$

Similarly, the surface charge density in the aqueous side of the EDL $Q_\mathrm{w} = F\int_0^{\infty}\left[c_{\mathrm{H}^+} + 4c_{\mathrm{Ce}^{4+}} - 2c_{\mathrm{SO}_4^{2-}}\right]\mathrm{d}x = (\epsilon_0\epsilon_{\mathrm{r,w}}/f)\mathrm{d}\varphi/\mathrm{d}x|_{x\to 0+}$ satisfies

$$\frac{Q_\mathrm{w}^2}{2\epsilon_0 RT} = \epsilon_{\mathrm{r,w}}c_{\mathrm{b,w}}\left[(1 + 2r_{\mathrm{Ce}})\mathrm{e}^{2[\varphi(0) - f\Delta_\mathrm{o}^\mathrm{w}\phi]} + 2\mathrm{e}^{-[\varphi(0) - f\Delta_\mathrm{o}^\mathrm{w}\phi]} + r_{\mathrm{Ce}}\mathrm{e}^{-4[\varphi(0) - f\Delta_\mathrm{o}^\mathrm{w}\phi]} - 3 - 3r_{\mathrm{Ce}}\right], \tag{S11}$$

where $r_{\mathrm{Ce}} = c_{\mathrm{b,Ce}}/c_{\mathrm{b,w}} = 10^{-2}$ and $\epsilon_{\mathrm{r,w}}$ is the relative permittivity of the aqueous solution.

The condition of global electroneutrality of the EDL is $Q_\mathrm{w} = -Q_\mathrm{o}$, Equation S4. From Equations S10 and S11, the equivalent condition $Q_\mathrm{o}^2/2\epsilon_0 RT = Q_\mathrm{w}^2/2\epsilon_0 RT$ can be solved to obtain the dimensionless interfacial potential $\varphi(0)$ for any applied interfacial potential difference $\Delta_\mathrm{o}^\mathrm{w}\phi = \phi^\mathrm{w} - \phi^\mathrm{o}$. The potential drops in the phases o and w are $\phi(0) - \phi^\mathrm{o} = \varphi(0)/f$ and $\phi^\mathrm{w} - \phi(0)$, respectively. Approximately 90 % of the applied potential resides in phase o (Figure S3). At the positive end of the polarisable potential window (PPW) at $\Delta_\mathrm{o}^\mathrm{w}\phi$ = +0.50 V, the potential drop in phase o is $\phi(0) - \phi^\mathrm{o}$ = 0.458 V. At the negative end of the PPW at $\Delta_\mathrm{o}^\mathrm{w}\phi$ = –0.35 V, the potential drop in phase o is $\phi(0) - \phi^\mathrm{o} = -0.320$ V. This significant difference between $\phi(0) - \phi^\mathrm{o}$ and $\phi^\mathrm{w} - \phi(0)$ is mostly due to the difference in relative permittivities, and to a lesser extent to the difference in electrolyte concentrations.

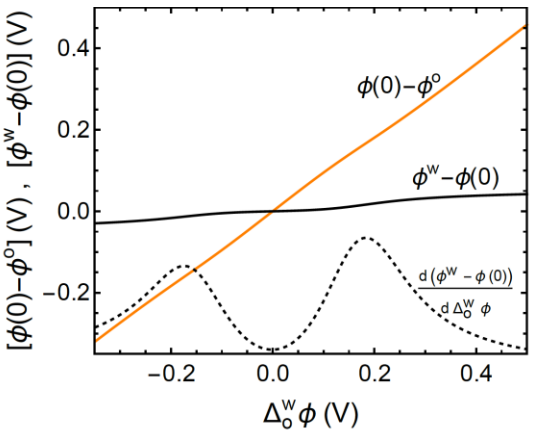

Figure S3. The applied potential $\Delta_o^w\phi$ is the sum of the potential drop $\phi(0)-\phi^o$ in phase o and the potential drop $\phi^w-\phi(0)$ in phase w. The dashed curve represents the rate (in arbitrary units) of the variation of the potential drop in phase w with the applied potential. It shows two maxima around –0.2 V and +0.2 V, which are related to the steric effects (*i.e.*, finite size of the organic electrolyte ions).

The contribution of BA^+ ions to the surface charge density in the organic side of the EDL can be calculated for any $\Delta_o^w\phi$ as

$$Q_{BA^+} = F\int_{-\infty}^{0}\left[c_{BA^+}(x) - c_{b,o}\right]dx = Fc_{b,o}\int_0^{\varphi(0)}\left[\frac{e^{-\varphi}}{1+\phi_{o,steric}(\cosh\varphi - 1)} - 1\right]\frac{d\varphi}{d\varphi/dx}$$

$$= -\frac{Fc_{b,o}}{\kappa_o}\left(\frac{\phi_{o,steric}}{2}\right)^{1/2}\int_0^{\varphi(0)}\frac{1-\frac{e^{-\varphi}}{1+\phi_{o,steric}(\cosh\varphi-1)}}{\{\ln[1+\phi_{o,steric}(\cosh\varphi-1)]\}^{1/2}}d\varphi \qquad \text{(S12)}$$

by numerical evaluation of the integral. The contribution of TB^- ions $Q_{TB^-} = Q_o - Q_{BA^+}$, where Q_o is given by Equation S10. The contributions of the different ions to the surface charge density in the aqueous side of the EDL can be calculated similarly.

The charge density in the organic side of the EDL is basically due to the accumulation ($c_i(x) > c_i^b$) of BA^+ or TB^- ions, because the bulk concentration $c_{b,o}$ = 5 mM is so small that ion depletion ($c_i(x) < c_i^b$) is a minor contribution. On the contrary, since $c_{b,w}$ = 200 mM H_2SO_4

is significantly larger, the charge density in the aqueous side of the EDL is due to both the accumulation of one supporting electrolyte ionic species and the depletion of the other. For instance, when $\Delta_o^w\phi$ = +0.20 V, the charge density is Q_w = 3.321 μC·cm^{-2}, of which 42.0 % is due to H^+ accumulation and 50.4 % to SO_4^{2-} depletion; note that divalent ions are more sensitive and responsive to the field at the EDL. The other 7.6 % is due to Ce^{4+} accumulation. It is interesting to observe that the Ce^{4+} contribution becomes significant from $\Delta_o^w\phi \approx$ +0.20 V and increases more rapidly with increasing $\Delta_o^w\phi$ than the H^+ contribution because Ce^{4+} is tetravalent (Figure S4a).

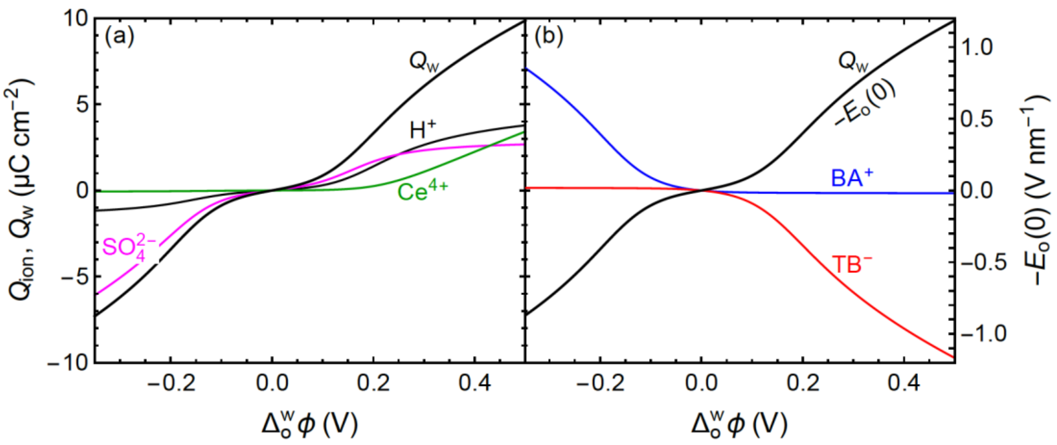

Figure S4. The charge density $Q_w = -Q_o$ separated across the L|L interface increases with $\Delta_o^w\phi$ for the cell configuration described in Figure 1a (in the absence of EDOT). This charge density is a sum of contributions from **(a)** aqueous and **(b)** organic ions, *i.e.*, $Q_w = Q_{H^+} + Q_{Ce^{4+}} + Q_{SO_4{}^{2-}} = -Q_{BA^+} - Q_{TB^-} = -Q_o$. The thick black curve represents both Q_w in the aqueous side of the EDL (left axis) and the negative electric field $-E_o$ (right axis) at the organic side of the interface x = 0–, as they are proportional to each other.

The electric displacement vector is continuous at the interface, a condition equivalent to $Q_\mathrm{w} = -Q_\mathrm{o}$. Since the relative permittivity $\epsilon_\mathrm{r,o} = 9.40$ of TFT is smaller than that of water ($\epsilon_\mathrm{r,w}$ = 78), the electric field is larger on the organic side of the interface. The intense electric field E_o at the organic side of the interface (Figure S4b) may be relevant for the orientation of dipoles, such as neutral EDOT (with a dipole moment of 1.87 D) when they are present.[12]

The slope of the total Q_w vs. $\Delta_\mathrm{o}^\mathrm{w}\phi$ curve is the differential capacitance (shown in Figure S6d, *vide infra*). It is already clear in Figure S4 that this slope shows two maxima around –0.2 V and +0.2 V. These maxima are related to the steric effect of the organic ions, as evidenced by the representation of their concentrations at the interface *x* = 0 in Figure 3a and Figure S5. Since the electric potential drop $\phi^\mathrm{w} - \phi(0)$ in the aqueous side of the EDL is relatively small, the interfacial ionic concentrations can be estimated with the Boltzmann equation without reaching unrealistic values. Note that the interfacial concentration of the oxidant Ce^{4+} is larger than 1 M for $\Delta_\mathrm{o}^\mathrm{w}\phi$ > 0.44 V, see Figure 3a. On the contrary, since the potential drop $\phi(0) - \phi^\mathrm{o}$ in the organic phase is very large (Figure S3), the Boltzmann equation would predict unrealistic values of the interfacial concentrations of the organic ions BA^+ or TB^-. Hence, the use of a modified Boltzmann equation with a maximum ionic concentration, to account for the steric effect, is needed. In Figure 3a and Figures S3 and S4, we have used $c_\mathrm{o,max}$ = 2 M as a tentative value and observe that the maximum organic ion concentration is reached when $\Delta_\mathrm{o}^\mathrm{w}\phi \approx \pm 0.2$ V. In order to show that the qualitative features are not so sensitive to the value of $c_\mathrm{o,max}$, in Figure S5 only we use a smaller value, $c_\mathrm{o,max}$ = 1 M. Should we compare the values of the applied $\Delta_\mathrm{o}^\mathrm{w}\phi$ at which the organic ions BA^+ or TB^- reach a concentration close to $c_\mathrm{o,max}$, it would be observed that $c_\mathrm{o,max}$= 1 M is reached at lower $\Delta_\mathrm{o}^\mathrm{w}\phi$ than $c_\mathrm{o,max}$ = 2 M. That is, the maxima around –0.2 V and +0.2 V in the dotted curve of Figure S3 ($c_\mathrm{o,max}$ = 2 M) would be shifted closer to 0 V by ca. 18 mV $\approx (RT/F)\ln(2\ \mathrm{M}/1\ \mathrm{M})$ if a

similar curve is plotted for the case $c_{o,max}$ = 1 M. Thus, the relevant feature is the appearance of maxima due to finite ion sizes, and the variation of the value of $c_{o,max}$ between 1 M and 2 M only shifts the location of the maxima by less than 18 mV. Hence, we conclude that 1 M or 2 M is a reasonable value for $c_{o,max}$. This conclusion can be further supported from the effective diameter of the BA^+ and TB^- ions, which can be estimated[13] as $a \approx 0.98$ nm, and a simple cubic packing density[14] $c_{o,max} \approx 1/(N_A a^3) \approx 1.8$ M, where N_A is Avogadro's constant.

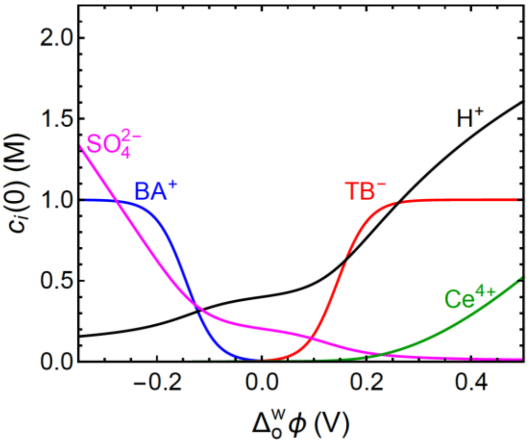

Figure S5. Interfacial ionic concentrations $c_i(0)$, evaluated using Equations S6 and S7 at $x = 0$, for the electrochemical cell configuration described in Figure 1a (in the absence of EDOT) as a function of the applied $\Delta_o^w\phi$. The maximum concentration of BA^+ and TB^- was $c_{o,max}$ = 1 M and the potential of zero charge (PZC) was set at 0 V on the Galvani scale.

It is worth clarifying that the interfacial ionic concentrations in Figures 3a and S5 are with reference to free ions and not salt concentrations as is the case in the bulk aqueous and organic electrolyte solutions. Thus, it is possible to reach molar concentrations of Ce^{4+} in the aqueous side of the EDL, or BA^+ or TB^- in the organic side of the EDL, by modulating the applied $\Delta_o^w\phi$. Such molar concentrations far exceed the solubilities of $Ce(SO_4)_2$ in water and

BATB in TFT. Thus, using a polarisable aqueous|organic interface opens opportunities to carry out chemical reactions, such as the redox reaction between Ce^{4+} and EDOT, in a local (interfacial) environment simply not attainable at a non-polarisable L|L interface or by dissolving the salts in either bulk phase. Additionally, advantage can be taken of the intrinsic property of polarisable L|L interfaces to accumulate neutral species, enabling a decrease in the (often expensive) monomer concentration in the bulk organic phase. For example, herein 5 mM EDOT was dissolved in the bulk TFT during interfacial electrosynthesis, whereas conventional electropolymerisation at solid electrode|electrolyte interfaces requires at least 100 mM to avoid overoxidation.

A strong validation of this model is the clear agreement between the features of the CV (Figure S6a) and differential capacitance curve (Figure S6c) experimentally observed and the modelled features of the CV (Figure S6b) and differential capacitance (Figure S6d) due to the capacitive current for the electrochemical cell configuration described in Figure 1a (in the absence of EDOT) as a function of the applied $\Delta_o^w \phi$. Note that while the PZC for the model is arbitrarily set at 0 V, the experimentally determined PZC was found to be ca. 0.1 V. The two maxima observed for the CVs and differential capacitance curves in both the experimental and modelled data are due to the finite size of the organic ions.

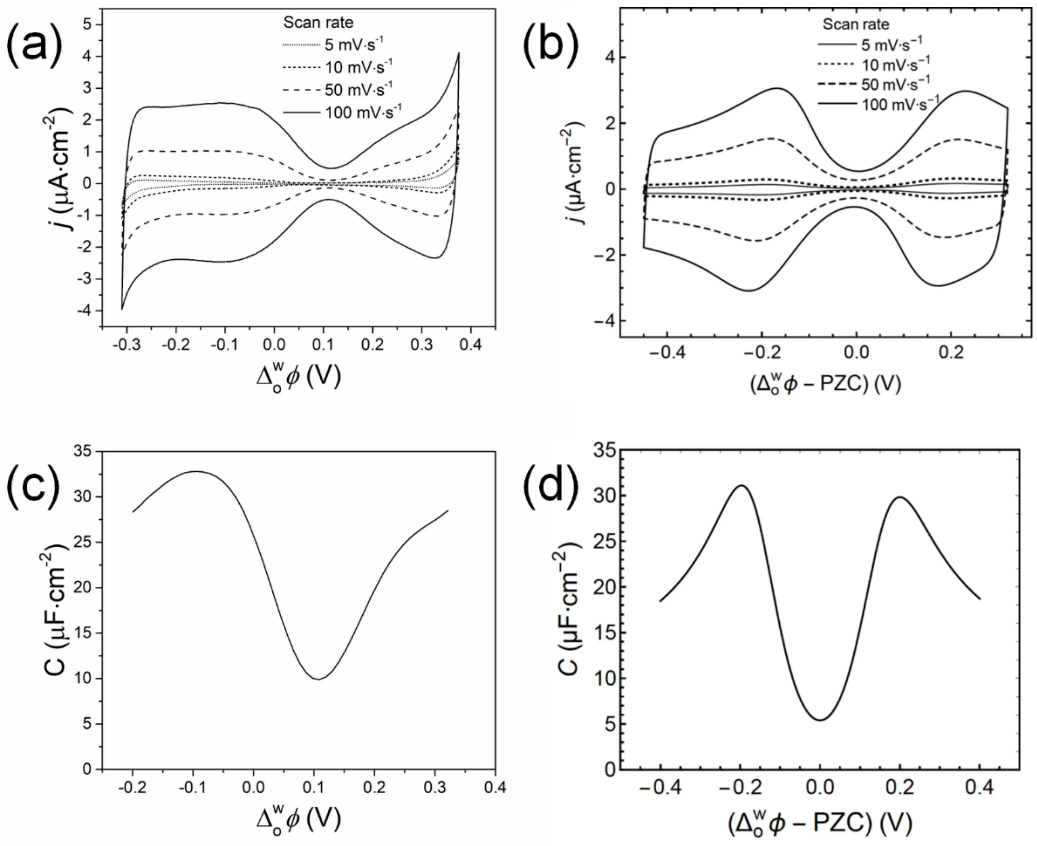

Figure S6. Comparison of experimental and modelled CVs and differential capacitance curves for the electrochemical cell configuration described in Figure 1a (in the absence of EDOT) as a function of the applied $\Delta_{\mathrm{o}}^{\mathrm{w}}\phi$. **(a)** Experimental CVs at increasing scan rates from 10 to 100 mV·s^{-1}. **(b)** Modelled CVs based on purely capacitive currents at the same scan rates using R_{sol}=0.5 Ω m^2 and R_{ct}=1.0 kΩ m^2. **(c)** Experimental differential capacitance measured at a frequency of 10 Hz with an amplitude of 10 mV. The scan direction was from positive to negative $\Delta_{\mathrm{o}}^{\mathrm{w}}\phi$. **(d)** Modelled differential capacitance measurement for $c_{\mathrm{o,max}} = 2$ M. The slope of the total Q_{w} curve (shown in Figure S4) is the differential capacitance.

Section S5. Spectroelectrochemical UV-vis-NIR analysis of a PEDOT thin film

To examine the electrochemically induced doping (both *p*- and *n*-doping) and de-doping of a PEDOT thin film, the latter was carefully extracted from the L|L interface after preparation by interfacial electrosynthesis and re-immobilised on the surface of a polished glassy carbon electrode. Cyclic voltammetry was performed in a solution of 0.1 M tetrabutylammonium hexafluorophosphate ($TBAPF_6$) in acetonitrile, which was dried with molecular sieves to remove any water present and filtered. Before the experiment, the electrolyte solution was bubbled with N_2 for 30 mins to remove O_2 from the electrochemical cell. The first cycle (red line) has several interesting features that are common in conducting polymer films (Figure S7a). At point (1), the increase in cathodic current corresponds to the reduction of the PEDOT thin film from the conductive oxidised to the insulating neutral state. The subsequent decrease in capacitive current is due to the de-doping of the PEDOT thin film and removal of the SO_4^{2-} counter anions from the polymer. When the potential reaches point (2), a cathodic peak is seen due to the release of trapped anions and solvent inside the PEDOT that takes place at the start of the *n*-doping process.[15,16] The reduction and *n*-doping of the PEDOT thin film begins at potentials close to –1.8 V (*vs.* Ag wire).[16] On the forward scan, the anodic current at point (3) corresponds to the oxidation and *p*-doping of the PEDOT film. This time, the positive charge in the PEDOT thin film is likely to be compensated by the PF_6^- electrolyte anion. The intense anodic peak observed after the onset of oxidation is frequently seen during the first CV cycle and is termed “the memory effect”. It signifies that when the PEDOT film is reduced, compaction or shrinkage of the polymer matrix takes place. As a result, additional energy is required to reopen the polymer film during oxidation and the *p*-doping.

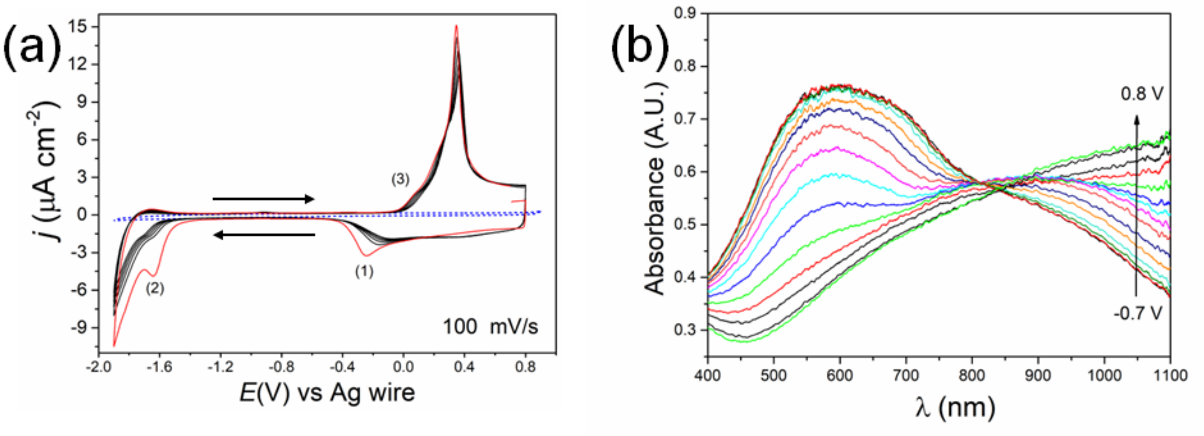

Figure S7. (a) CVs of a PEDOT thin film immobilised on the surface of a glassy carbon working electrode in 0.1 M $TBAPF_6$ in acetonitrile solvent, at a scan rate of 100 mV·s^{-1}. The red line shows the initial voltammogram, and the black line shows the subsequent cycles. The blue-dashed line shows the blank response of the glassy carbon electrode. The electrochemical cell was maintained under a N_2 for the duration of the experiment. The thickness of the PEDOT thin film under study was ~100 nm. A silver wire was used as *pseudo*-reference electrode. **(b)** UV-vis-NIR analysis of a PEDOT thin film re-immobilised on a transparent a two-strip Au interdigitated microarray electrode (NanoSPR, USA) in a solution of 0.1 M $TBAPF_6$ in acetonitrile at a range of potentials from –0.7 to +0.8 V to (*vs.* Ag wire). UV-vis-NIR spectroscopy measurements were performed using a home-made optical setup with a Maya 2000 Pro Spectrometer and deuterium – halogen lamp (DH2000) light source (Ocean Optics, USA). Absorbance measurements were taken after each potential was held (potentiostatically) for approximately 60 seconds. The potential was stepped sequentially from –0.7 to +0.8 V (*vs.* Ag wire). The spectrometer was controlled with OceanView software, version 1.6.7.

The spectroelectrochemical UV-vis-NIR response of a PEDOT thin film across a range of potentials from –0.7 to +0.8 V (*vs.* Ag wire) was probed in a solution of 0.1 M $TBAPF_6$ in acetonitrile by re-immobilising a PEDOT thin film on a transparent a two-strip Au

interdigitated microarray electrode (NanoSPR, USA), see Figure S7b. The spectra obtained were fully in line with previous spectroelectrochemical UV-vis-NIR studies of PEDOT in acetonitrile,[17,18] aqueous,[19] and room temperature ionic liquid[20] solutions. The neutral PEDOT thin film at –0.7 V (*vs.* Ag wire) shows a broad band centred around 580 nm that is associated with the π– π* transition and a weaker band around 890 nm. As the applied potential shifts positively, the π– π* band decreases continuously in intensity, whereas the band at 890 nm grows in intensity. The latter is associated with *p*-doping of the PEDOT thin film and ascribed to polaron type carriers. An isosbestic point observed at 800 nm is indicative of the gradual transformation of the insulating neutral phase into the conductive *p*-doped phase. At applied potentials >+0.4 V (*vs.* Ag wire), *i.e.,* beyond the potential corresponding to the end of the first anodic peak in Figure S7a, an increase in the NIR region of the spectrum at 1100 nm can be seen that overlaps the band at 890 nm. This band has been assigned to the evolution of bipolaron states within the PEDOT thin film. Furthermore, at applied potentials >+0.4 V (*vs.* Ag wire) the UV-vis-NIR curve no longer passes through the isosbestic point around 800 nm. This has been rationalised to be due to the transformation of the *p*-doped phase into a new "overdoped" phase which gives rise to a new isosbestic point around 850 nm.[18]

Section S6. Capacitive currents contributing to the steady growth of the charge accumulated during interfacial electrosynthesis

Numerous processes may lead to capacitive currents that contribute to the charge accumulated as the PEDOT thin film grows. Capacitive current may arise due to the polarisability of the PEDOT thin film under the influence of an applied external electric field. The PEDOT thin film is a dielectric material,[21–24] and the electric field induces charge separation within the film and self-organisation of nanodomains, thereby increasing the capacitance. Capacitive current may also arise due to accumulation of counteranions (either SO_4^{2-} and TB^-), possibly forming atomistic Stern layers, in the vicinity of charged cationic PEDOT chains and/or EDOT oligomers.[25–28] In other words, electrostatic molecular double layers defined at atomistic scales are formed between holes, localised in the PEDOT backbone, and their counterions.[29] The Stern layers have a purely electrostatic origin as the counterions do not form bonds with atoms of the PEDOT chains and charge transfer does not occur between the counterions and the PEDOT chains. As the thickness of the PEDOT thin film increases with repetitive CV cycling, the volumetric capacitance due to charges stored in the atomistic Stern layers formed by the PEDOT chains and/or EDOT oligomers and their counterions also increases.

Section S7. Modelling of peak β as capacitive current due to anion exchange

The capacitive current density j is positive when positive charge moves from phase w to phase o,

$$j = \frac{dQ_w}{dt} = -\frac{dQ_o}{dt} = \nu\sigma \frac{dx_o}{d\Delta_o^w\phi}, \tag{S13}$$

where $\nu \equiv d\Delta_o^w\phi/dt$ is the scan rate (positive in a forward scan and negative in a reverse scan) and $x_o = -Q_o/\sigma$. That is, j is determined by the variation with the applied $\Delta_o^w\phi$ of the concentrations of the ions that accumulate in region IN. The distribution of the ions between their respective bulk phases and the interphase region is affected by the potential drops between this region and the bulk phases, $\Delta_o^{IN}\phi = \phi^{IN} - \phi^o$ and $\Delta_w^{IN}\phi = \phi^{IN} - \phi^w$. Their relationship with $\Delta_o^w\phi = \phi^w - \phi^o$ is

$$\Delta_o^w\phi = \Delta_o^{IN}\phi - \Delta_w^{IN}\phi . \tag{S14}$$

It is apparent that $x_o = 1 - x_w$ increases when x_w decreases. Similarly, when $\Delta_o^w\phi$ is increased, $\Delta_o^{IN}\phi$ increases and $\Delta_w^{IN}\phi$ decreases. An increase in $\Delta_o^{IN}\phi$ promotes the accumulation of negative charge in region IN due to organic ions. Then, $d\Delta_o^w\phi > 0$ implies $dQ_o < 0$ and $dx_o = -(1/\sigma)dQ_o > 0$; as well as $dQ_w > 0$ and $dx_w < 0$. Since $dx_o/d\Delta_o^w\phi > 0$, the current density in Equation S13 has the same sign as the scan rate ν. The increase of $\Delta_o^w\phi$ from large negative values (with respect to the peak labelled β in Figures 1d–e) to large positive values corresponds to increasing x_o from 0 to 1. Hence, the area under the capacitive peak is $\int_{peak} j d\Delta_o^w\phi = \nu\sigma$.

The organic phase has a concentration c_b^o of the supporting electrolyte BATB and the aqueous phase has a concentration c_b^w of a strong acid H_zA that dissociates into protons H^+ and anions A^{z-}, where $z > 0$. We consider the cases $-z = -1$ and -2. The evaluation of Q_o and Q_w is simple if we restrict to applied potentials around the capacitive peak. With this

restriction, the concentrations in region IN of ionic species other than the organic anions TB^{-} and the aqueous anions A^{z-} can be neglected, *i.e.,* $Q_o \approx -Fc_{TB}^{IN}d$ and $Q_w \approx -zFc_A^{IN}d$ when $c_{BA}^{IN} \ll c_{TB}^{IN}$ and $c_H^{IN} \ll c_A^{IN}$. We assume that there is a distribution equilibrium of the anions between their bulk phases and the interphase region. The equilibrium conditions are

$$c_{TB}^{IN} = K_{TB}c_b^o e^{f\Delta_o^{IN}\phi} \tag{S15}$$

$$c_A^{IN} = K_A c_b^w e^{zf\Delta_w^{IN}\phi}, \tag{S16}$$

where $f = F/RT$ and K_{TB} (K_A) is the chemical partition coefficient of the organic anions TB^{-} (aqueous anions A^{z-}).

The fractions of the EDOT charge density that are compensated by the organic and aqueous anions are $x_o \equiv -Q_o/\sigma \approx c_{TB}^{IN}/c_{EDOT}^{IN}$ and $x_w = 1 - x_o \approx zc_A^{IN}/c_{EDOT}^{IN}$, respectively. The distribution of the anions in the interphase region IN is modulated by the applied $\Delta_o^w\phi$. The fraction x_o is also determined by the partition coefficients K_{TB} and K_A, which in turn determine the peak potential $\Delta_o^w\phi_p$. An increase in $\Delta_o^{IN}\phi$ promotes the accumulation of TB^{-} anions in region IN. Then, $d\Delta_o^w\phi > 0$ implies $dQ_o < 0$ and $dx_o = -(1/\sigma)dQ_o > 0$; as well as $dQ_w > 0$ and $dx_w < 0$. Since $dx_o/d\Delta_o^w\phi > 0$, the current density in Equation S13 has the same sign as the scan rate ν.

Eliminating $\Delta_o^{IN}\phi$ and $\Delta_w^{IN}\phi$ between Equations S14–S16, we obtain that the relation between $x_o \approx c_{TB}^{IN}/c_{EDOT}^{IN}$ and $\Delta_o^w\phi$ is

$$\frac{x_o^z}{1-x_o} = \left(\frac{c_{TB}^{IN}}{c_{EDOT}^{IN}}\right)^z \frac{c_{EDOT}^{IN}}{zc_A^{IN}} = \frac{(K_{TB}c_b^o)^z}{zK_A c_b^w (c_{EDOT}^{IN})^{z-1}} e^{zf\Delta_o^w\phi} \ . \tag{S17}$$

In terms of the dimensionless applied potential

$$\varphi \equiv zf(\Delta_o^w\phi - \Delta_o^w\phi_p) \ , \tag{S18}$$

the peak corresponds to $\varphi = 0$ and Equation S17 transforms to

$$\zeta(z)\frac{x_o^z}{1-x_o} = e^{\varphi} \ , \quad \text{(S19)}$$

where $\zeta(z) \equiv (1-x_{o,p})/x_{o,p}^z$ and $x_{o,p}$ is the value (to be determined) of x_o at the peak. Equation S19 describes that the EDOT charge is compensated by organic anions when φ is large and positive and by aqueous anions when φ is large and negative, see Figure 5a(i). At $\varphi = 0$, the rate of anion exchange is maximum. Since j is proportional to $dx_o/d\varphi$, the peak appears when $d^2\varphi/dx_o^2 = 0$; because $d^2x_o/d\varphi^2$ vanishes when $d^2\varphi/dx_o^2$ does. Taking the logarithm of Equation S19 and deriving it twice with respect to x_o it is concluded that $d^2\varphi/dx_o^2 = 0$ when x_o takes the value $x_{o,p} = 1/(1+1/\sqrt{z})$ and, therefore $\zeta(z) = (1+1/\sqrt{z})^z/(1+\sqrt{z})$. From Equations S17–S19, the peak potential is

$$\Delta_o^w\phi_p \equiv -\frac{RT}{zF}\ln\left[\frac{\zeta(z)\,(K_{TB}c_b^o)^z}{zK_A c_b^w (c_{EDOT}^{IN})^{z-1}}\right] \quad \text{(S20)}$$

and from Equations S13 and S20 the peak current density is

$$j_p = zfv\sigma\left.\frac{dx_o}{d\varphi}\right|_{peak} = \frac{zfv\sigma}{(1+\sqrt{z})^2} \ . \quad \text{(S21)}$$

The peak current density is $j_{p1} = fv\sigma/4$ for monovalent anions and $j_{p2} = 0.3431fv\sigma$ for divalent anions. For constant sweep rate v and EDOT charge density σ, the area under the capacitive peak does not change with z, $\int_{peak}(j/j_{p1})d\Delta_o^w\phi = 4/f \approx 0.103$ V, see Figure 5b(ii).

For monoprotic aqueous acids ($-z = -1$), Equation S19 is similar to a Fermi-Dirac distribution,[30]

$$1 - x_o = x_w = \frac{1}{e^{\varphi}+1} \quad \text{(S22)}$$

and the current density due to anion exchange is

$$j = 4j_{p1}x_o(1-x_o) = \frac{j_{p1}}{\cosh^2[f(\Delta_o^w\phi - \Delta_o^w\phi_p)/2]} \quad (-z = -1)\ . \quad \text{(S23)}$$

As expected from Equation S22, Equation S23 describes a symmetric peak, with $x_{\mathrm{o,p}} = x_{\mathrm{w,p}} = 1/2$ and $\zeta(1) = 1$, see Figure 5b(ii) (blue curve). On the contrary, the peak is asymmetric for divalent aqueous anions ($-z = -2$), with $1 - x_{\mathrm{o,p}} = x_{\mathrm{w,p}} = 1/(1+\sqrt{2}) \approx 0.4142$ and $\zeta(2) = (1+\sqrt{2})/2 \approx 1.207$. This asymmetric peak can be represented in parametric form $(\varphi(x_{\mathrm{o}}), j(x_{\mathrm{o}}))$, with

$$j = 2f\nu\sigma\frac{\mathrm{d}x_{\mathrm{o}}}{\mathrm{d}\varphi} = 8j_{\mathrm{p1}}\frac{x_{\mathrm{o}}(1-x_{\mathrm{o}})}{2-x_{\mathrm{o}}} \quad (-z = -2) \qquad \text{(S24)}$$

and $\varphi(x_{\mathrm{o}}) = \ln[\zeta(2)x_{\mathrm{o}}^2/(1-x_{\mathrm{o}})]$, see Figure 5b(ii) (black curve). The shape of this peak is in very good agreement with the shape of the peak β experimentally observed when using H_2SO_4 as the aqueous electrolyte, see Figure 1d.

Section S8. Modelling the capacitive current peak due to proton adsorption

Using the electrochemical cell configuration shown in Figure S8a, pH dependent reversible proton adsorption/desorption to an immobilised PEDOT thin film at the polarisable aqueous|TFT interface is observed experimentally (Figure S8b and described in the main text). The latter is purely electrostatic in nature with no chemical bonding occurring when protons adsorb from the aqueous phase. Instead, the charge of the protons is compensated by adsorbed organic TB^- species as modelled in this section.

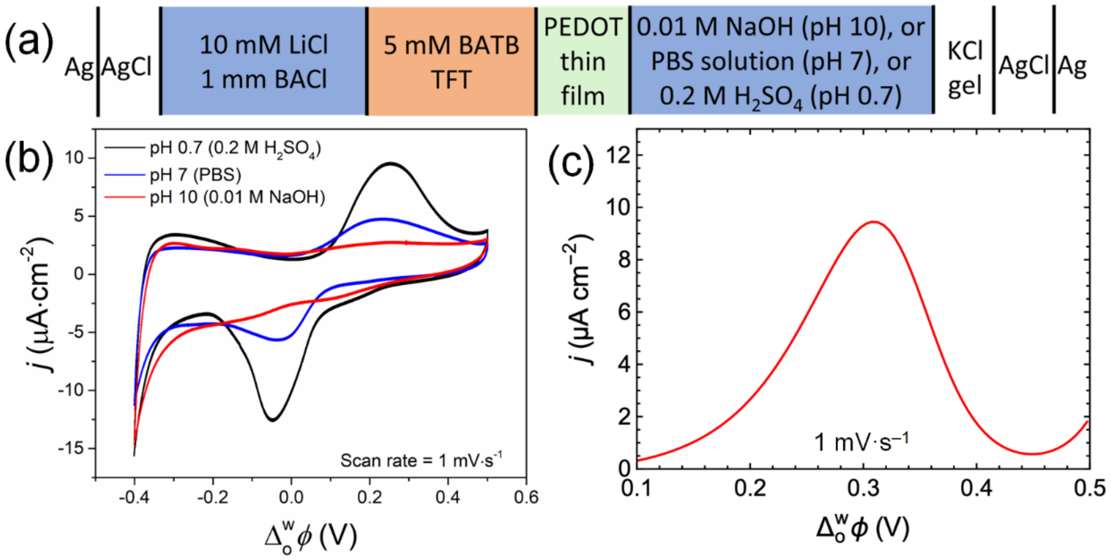

Figure S8. Electrochemical responses of a PEDOT thin film re-immobilised on a polarisable aqueous|TFT interface as a function of aqueous phase pH. (a) Configuration of the four-electrode electrochemical cell employed to probe the pH dependent reversible adsorption/desorption of protons from the re-immobilised PEDOT thin film. **(b)** Cyclic voltammetry of re-immobilised PEDOT thin films as a function of the aqueous phase pH using the electrochemical cell configurations described in **(a)**. All CVs were recorded under ambient, aerobic conditions at a scan rate of 1 $mV \cdot s^{-1}$. **(c)** Model of the electrochemical response of a re-immobilised PEDOT thin film at 1 $mV \cdot s^{-1}$ using the cell configuration described in **(a)** under acidic conditions (*i.e.*, using 0.2 M H_2SO_4) on the forward scan at a positive applied $\Delta_o^w\phi$.

The organic phase has a concentration $c_\mathrm{b}^\mathrm{o} = 5$ mM of the supporting electrolyte BATB and the aqueous phase has a concentration $c_\mathrm{b}^\mathrm{w} = 200$ mM of H_2SO_4, which is assumed to dissociate into protons H^+ and SO_4^{2-}, for the sake of simplicity. For positive applied $\Delta_\mathrm{o}^\mathrm{w}\phi$, SO_4^{2-} anions stay in the aqueous phase and protons are electrically pushed into region IN, where they can adsorb to the sites in the PEDOT film. The surface charge density due to free and adsorbed protons in region IN is

$$Q_\mathrm{w} \approx F(c_\mathrm{H}^\mathrm{IN} + c_\mathrm{H,ad}^\mathrm{IN})d\ . \tag{S25}$$

Since protons adsorbed on different sites do not interact with each other, the Langmuir isotherm

$$c_\mathrm{H,ad}^\mathrm{IN} = c_\mathrm{site}^\mathrm{IN} \frac{\kappa_\mathrm{ad} c_\mathrm{H}^\mathrm{IN}}{1+\kappa_\mathrm{ad} c_\mathrm{H}^\mathrm{IN}} \tag{S26}$$

describes the occupation of the adsorption sites, where κ_ad is the adsorption constant. If we restrict to applied potentials around the peak potential $\Delta_\mathrm{o}^\mathrm{w}\phi_\mathrm{p} \approx 0.26$ V observed in the forward scan at 1 mV·s^{-1} and 0.2 M H_2SO_4 in Figure S8b, the global electroneutrality of region IN, Equation 3 (in the main text), reduces to

$$c_\mathrm{TB}^\mathrm{IN} = c_\mathrm{EDOT}^\mathrm{IN} + c_\mathrm{H}^\mathrm{IN} + c_\mathrm{H,ad}^\mathrm{IN}\ . \tag{S27}$$

Since the surface charge density due to organic anions TB^- that accumulate in region IN is $Q_\mathrm{o} \approx -F c_\mathrm{TB}^\mathrm{IN} d$, the capacitive current density is evaluated from Equation S13 as

$$j = -\frac{\mathrm{d}Q_\mathrm{o}}{\mathrm{d}t} = \nu F d \frac{\mathrm{d}c_\mathrm{TB}^\mathrm{IN}}{\mathrm{d}\Delta_\mathrm{o}^\mathrm{w}\phi}\ , \tag{S28}$$

where $\nu \equiv \mathrm{d}\Delta_\mathrm{o}^\mathrm{w}\phi/\mathrm{d}t$ is the scan rate ($\nu = 1$ mV·s^{-1} in the forward scan under consideration).

The distribution equilibrium of the organic anions and the aqueous protons between their bulk phases and the interphase region requires that

$$c_\mathrm{TB}^\mathrm{IN} = K_\mathrm{TB} c_\mathrm{b}^\mathrm{o} \mathrm{e}^{f\Delta_\mathrm{o}^\mathrm{IN}\phi} \tag{S29}$$

$$c_{\mathrm{H}}^{\mathrm{IN}} = K_{\mathrm{H}} c_{\mathrm{H,b}}^{\mathrm{w}} \mathrm{e}^{-f \Delta_{\mathrm{w}}^{\mathrm{IN}} \phi}, \tag{S30}$$

where $f = F/RT$, K_{TB} (K_{H}) is the chemical partition coefficient of the organic anions TB^- (aqueous protons H^+) and $c_{\mathrm{H,b}}^{\mathrm{w}} = 2c_{\mathrm{b}}^{\mathrm{w}}$. Eliminating $\Delta_{\mathrm{o}}^{\mathrm{IN}}\phi$ and $\Delta_{\mathrm{w}}^{\mathrm{IN}}\phi$ between Equations S14, S29 and S30, we obtain that

$$\frac{c_{\mathrm{TB}}^{\mathrm{IN}}}{K_{\mathrm{TB}} c_{\mathrm{b}}^{\mathrm{o}}} \frac{c_{\mathrm{H}}^{\mathrm{IN}}}{K_{\mathrm{H}} c_{\mathrm{H,b}}^{\mathrm{w}}} = \mathrm{e}^{f \Delta_{\mathrm{o}}^{\mathrm{w}} \phi} . \tag{S31}$$

Equations S26, S29 and S31 describe the variations of $c_{\mathrm{TB}}^{\mathrm{IN}}$ and $c_{\mathrm{H,ad}}^{\mathrm{IN}}$ with $\Delta_{\mathrm{o}}^{\mathrm{w}}\phi$. Essentially, Equation S26 describes that $c_{\mathrm{H,ad}}^{\mathrm{IN}}$ increases from 0 to $c_{\mathrm{site}}^{\mathrm{IN}}$ as $\Delta_{\mathrm{o}}^{\mathrm{w}}\phi$ is increased. The peak current corresponds approximately to half occupation of the adsorption sites, $c_{\mathrm{H,ad,p}}^{\mathrm{IN}} \approx c_{\mathrm{site}}^{\mathrm{IN}}/2$, which corresponds to $c_{\mathrm{H,p}}^{\mathrm{IN}} \approx 1/\kappa_{\mathrm{ad}}$. Thus, using $c_{\mathrm{TB,p}}^{\mathrm{IN}} \approx c_{\mathrm{H,ad,p}}^{\mathrm{IN}}$ in Equation S30, the peak potential is approximately given by $\Delta_{\mathrm{o}}^{\mathrm{w}}\phi_{\mathrm{p}} \approx (RT/F)\ln[c_{\mathrm{site}}^{\mathrm{IN}}/(2K_{\mathrm{TB}}K_{\mathrm{H}}\kappa_{\mathrm{ad}}c_{\mathrm{b}}^{\mathrm{o}}c_{\mathrm{H,b}}^{\mathrm{w}})]$. Moreover, the area under the peak is determined by the product of the concentration $c_{\mathrm{site}}^{\mathrm{IN}}$ of adsorption sites and the thickness d. Thus, for an estimation of the PEDOT thin film thickness of $d = 400\,\mathrm{nm}$, the values $c_{\mathrm{EDOT}}^{\mathrm{IN}} = 10\,\mathrm{mM}$ and $c_{\mathrm{site}}^{\mathrm{IN}} = 362\,\mathrm{mM}$ can be chosen to match the experimentally observed area under the adsorption peak. The values $K_{\mathrm{TB}} = 0.30$ and $K_{\mathrm{H}} = 3.0 \times 10^{-8}$, for the partition coefficients and $\kappa_{\mathrm{ad}} = 100\,\mathrm{mM}^{-1}$ for the adsorption constant give a peak potential very close to the observed $\Delta_{\mathrm{o}}^{\mathrm{w}}\phi_{\mathrm{p}} \approx 0.26\,\mathrm{V}$. The adsorption peak calculated with Equation S28 has an asymmetric shape (Figure S8c) that matches very well with the experimental one (Figure S8b, 0.2 M H_2SO_4). Note that we are not fitting the experimental data but this simulation strongly supports the interpretation that the peak is due to proton adsorption.

Section S9. Scan rate studies of PEDOT interfacial electrosynthesis

To probe the influence of scan rate on PEDOT thin film electrosynthesis, further CV cycling experiments using the electrochemical cell configuration described in Figure 1a were carried out at 1 and 100 $mV \cdot s^{-1}$. At the higher scan rate of 100 $mV \cdot s^{-1}$, PEDOT interfacial films were formed after 50 cycles using both 2 and 4 mM aqueous Ce^{4+} concentrations, respectively (Figures S9 and S10). With 4 mM Ce^{4+}, a nucleation loop was observed after seven cycles (Figure S10b). This indicates that, on the relatively short timescale during which the applied $\Delta_{\mathrm{o}}^{\mathrm{w}}\phi$ was scanned positive enough to induce IET, the concentration of EDOT^{n+} oligomers generated and undergoing comproportionation with EDOT monomers was low until the nucleation loop appeared. At that point (cycle 7), the autocatalytic reaction began, significantly boosting the rate of EDOT oxidation, leading to a higher generation of $\mathrm{EDOT}^{\bullet+}$ in the organic side of the EDL. As a result, the oligomer chains formed at 100 $mV \cdot s^{-1}$ reached the critical length to induce oligomer precipitation and film formation.

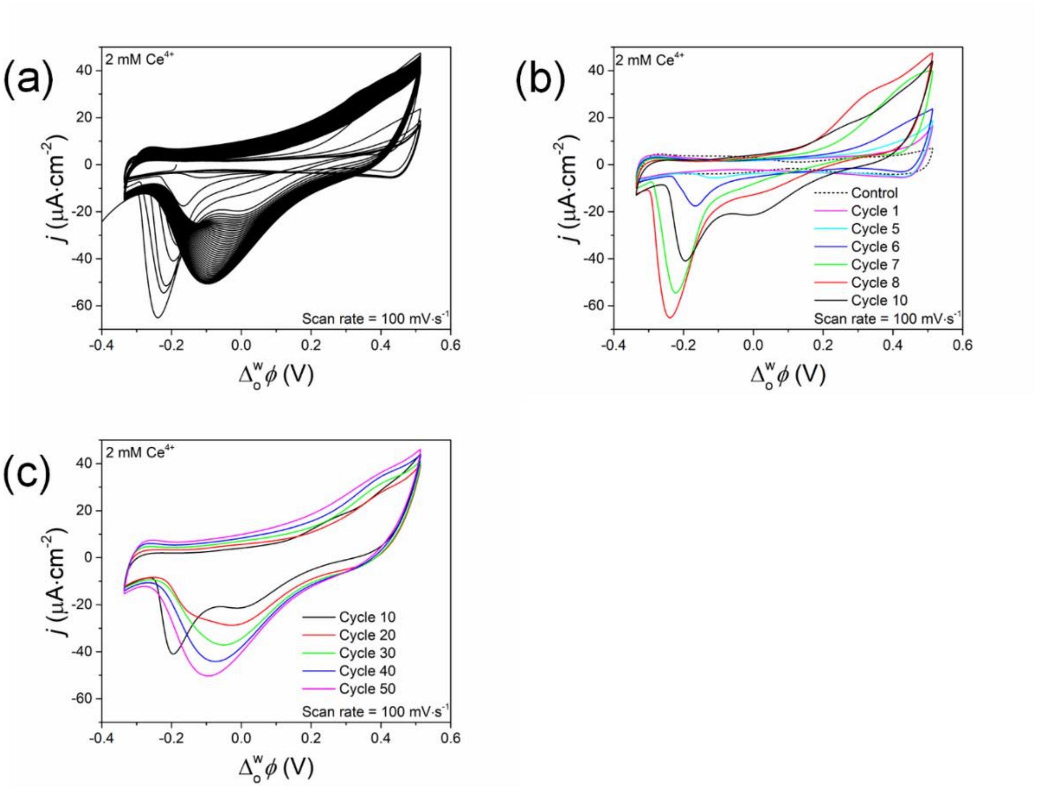

Figure S9. Interfacial electrosynthesis of a PEDOT thin film by repetitive CV cycling at a scan rate of 100 mV·s^{-1} <u>using 2 mM Ce^{4+}</u>. (a) Fifty repetitive CV cycles in total (black CVs) were recorded using the electrochemical cell configuration described in Figure 1a with 2 mM $Ce(SO_4)_2$ in the aqueous phase. Selected cycles **(b)** 1 to 10 and **(c)** 10 to 50 as interfacial electrosynthesis proceeds are shown for clarity. For control experiments, the $Ce(SO_4)_2$ concentration was 0 mM (dashed CV).

Previously, we provided evidence for such an autocatalytic mechanism. Using repetitive DPSCA cycles, we demonstrated that the charge due to IET (on the step where a positive $\Delta_o^w \phi$ of +0.4 V was applied) initially increases slowly for the first 5 cycles during IET between Ce^{4+} and EDOT monomers at the bare L|L interface. However, a rapid increase in charge was measured for the next 5 cycles.[7] Thus, using the insights from the nucleation loops in the CVs observed herein, we can now attribute the commencement of that increase in the

rate of PEDOT interfacial electrosynthesis with DPSCA to the initiation of the comproportionation reaction and associated rapid increase in $EDOT^{\bullet+}$ in the organic side of the EDL. Consequently, the oligomer chains that were formed, rapidly reached the critical length to induce oligomer precipitation and the IET reaction then proceeded by a bipolar-like mechanism.

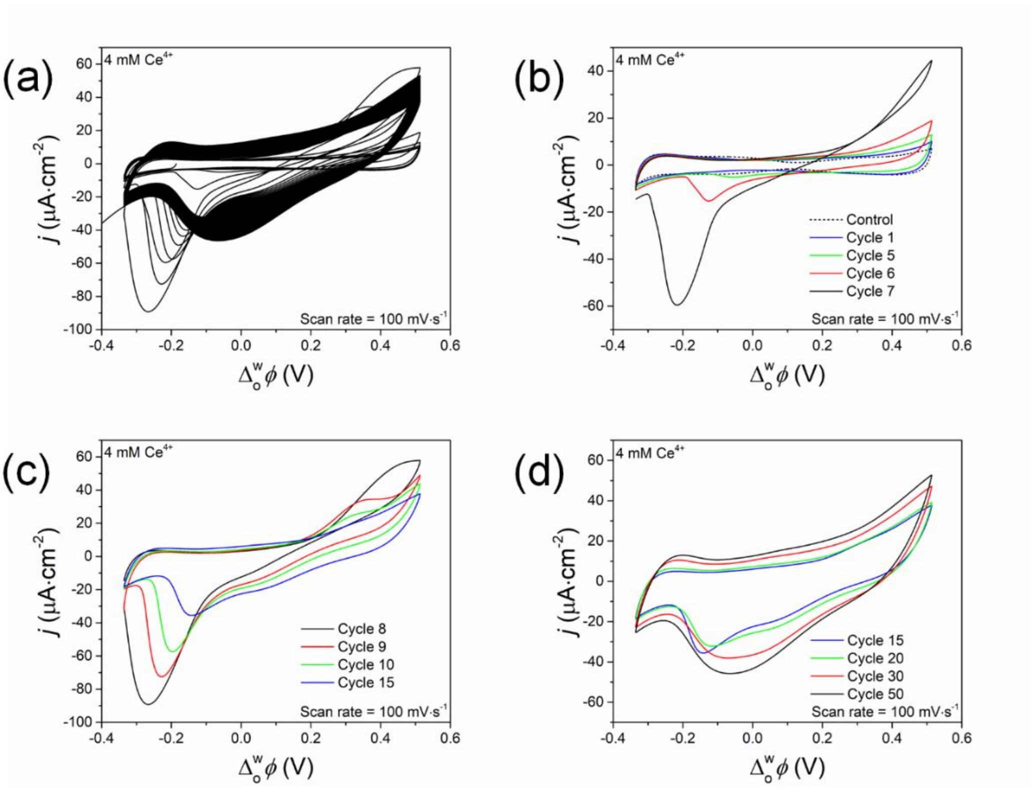

Figure S10. Interfacial electrosynthesis of a PEDOT thin film by repetitive CV cycling at a scan rate of 100 mV·s^{-1} using 4 mM Ce^{4+}. **(a)** Fifty repetitive CV cycles in total (black CVs) were recorded using the cell described in Figure 1a with 4 mM $Ce(SO_4)_2$ in the aqueous phase. Selected cycles **(b)** 1 to 7, **(c)** 8 to 15, and **(d)** 15 to 50 as interfacial electrosynthesis proceeds are shown for clarity. For control experiments, the $Ce(SO_4)_2$ concentration was 0 mM (dashed CV). A nucleation loop is observed during cycle 7 in **(b)**.

The PEDOT thin film formed at 100 mV·s^{-1} was notably thinner and had a more metallic or “golden” colour (Figure S11) than a PEDOT thin film formed at 25 mV·s^{-1} under otherwise identical conditions. A high concentration of EDOT oligomers is confined to a far thinner organic electrical double layer at 100 mV·s^{-1} than at 25 mV·s^{-1}, potentially leading to differences in the morphology and thickness of the film formed.

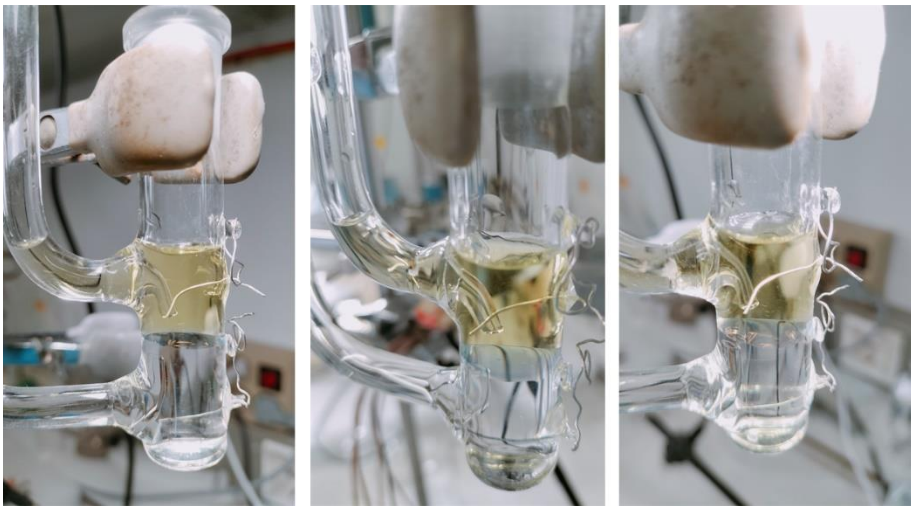

Figure S11. Images of the PEDOT thin film formed after 50 cycles with 4 mM Ce^{4+} at 100 mV·s^{-1} as described in Figure S10. The film is notably thinner than a film formed at 25 mV·s^{-1}, with a slightly greener colour and metallic “golden” shine.

At the lower scan rate of 1 mV·s^{-1}, no PEDOT interfacial film was formed after cycling using aqueous Ce^{4+} concentrations of either 2 or 6 mM, respectively (Figure S12). The CVs obtained using 2 mM Ce^{4+} remain unchanged with cycling, with no indication of IET taking place at positive applied $\Delta_o^w\phi$ (Figure S12a). At 1 mV·s^{-1}, the applied $\Delta_o^w\phi$ is scanned positively for a relatively long time, increasing the thickness of the organic diffuse layer and decreasing the local monomer and oligomer concentrations. The latter may inhibit the kinetics of IET. However, upon increasing the aqueous Ce^{4+} concentration to 6 mM, the characteristic

CVs of PEDOT interfacial electrosynthesis were observed during the initial cycles (Figure S12b) despite no PEDOT thin film forming. Clear nucleation loops were observed in the first and second cycles indicating the concentration of $EDOT^{n+}$ oligomers undergoing comproportionation with EDOT monomers was high, with a double nucleation loop seen in the second cycle (Figures S13a–b).

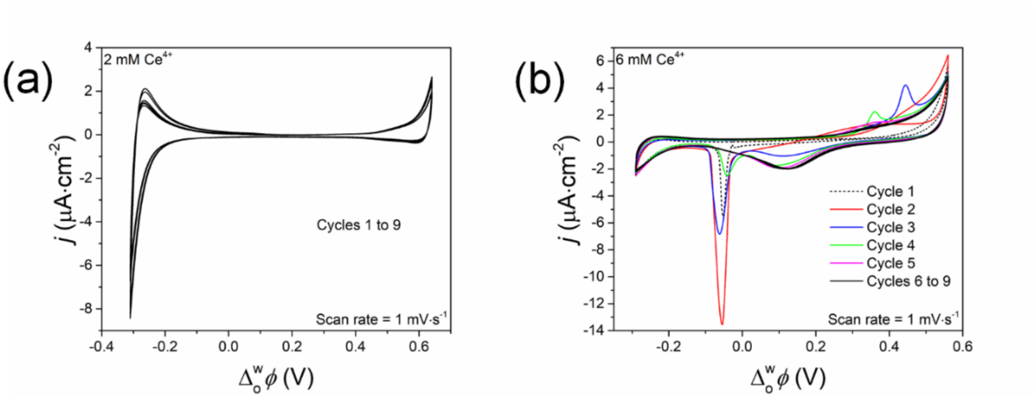

Figure S12. Interfacial electrosynthesis of a PEDOT thin film by repetitive CV cycling at a scan rate of 1 mV·s^{-1}. CVs were recorded, 9 repetitive CV cycles in total, using the electrochemical cell configuration described in Figure 1a with either **(a)** 2 mM or **(b)** 6 mM $Ce(SO_4)_2$ in the aqueous phase, respectively.

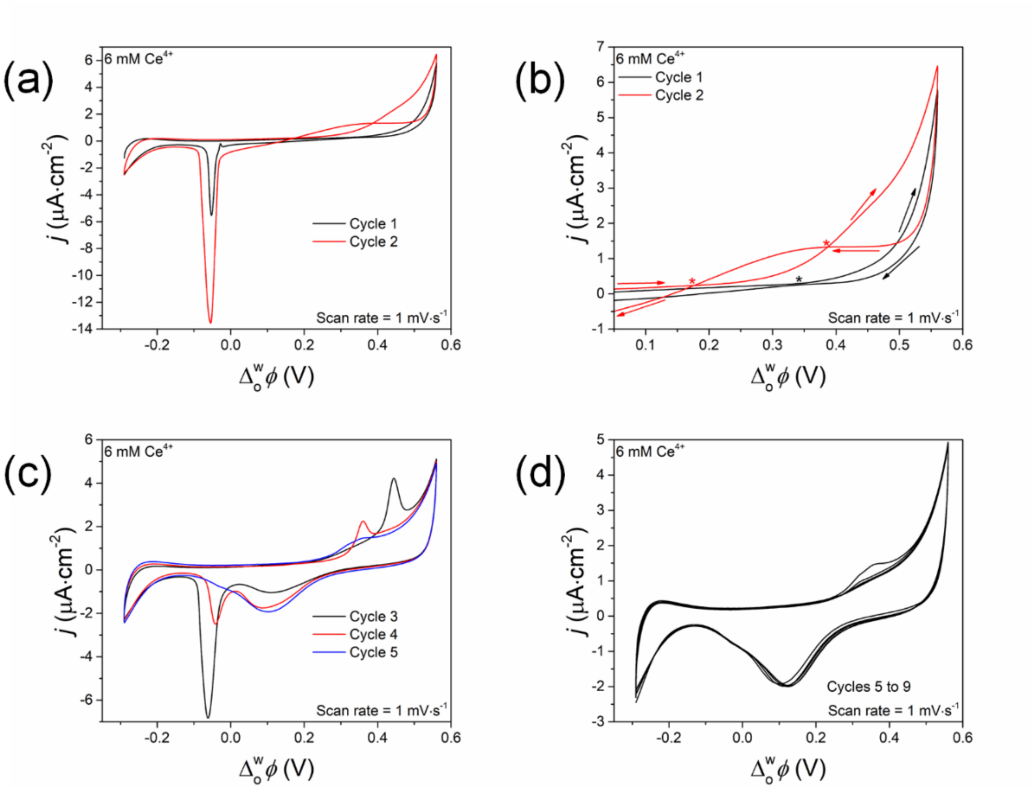

Figure S13. Interfacial electrosynthesis of a PEDOT thin film by repetitive CV cycling at a scan rate of 1 mV·s^{-1} using 6 mM Ce^{4+}. Nine repetitive CV cycles in total (as shown in Figure S12b) were recorded using the cell described in Figure 1a with 6 mM Ce(SO$_4$)$_2$. Selected cycles **(a, b)** 1 and 2, **(c)** 3 to 5, and **(d)** 5 to 9 as interfacial electrosynthesis proceeds are shown for clarity. In **(b)**, the black and red arrows indicate the scan direction for each CV and the red and black asterisk are cross-over points during the nucleation loops. During cycle 1, the absence of a diffusional peak and the exponential current (between 0.4 and 0.6 V) indicates that no depletion of reactants (Ce^{4+} and EDOT) occurs at 1 mV·s^{-1}. The appearance of the negative peak during the backward scan indicates the formation of oligomers during cycle 1. In **(c)**, during cycles 3 and 4, the positive peak associated with proton adsorption at positive applied $\Delta_o^w \phi$ is far more defined than at higher scan rates (25 and 100 mV·s^{-1}). In **(d)**, from cycle 5 to 9, proton adsorption/desorption is the most dominant feature. However, the absence of a steady build-up of charge with successive cycles indicates the electroactivity of the adsorbed species (shown to be overoxidised precipitate) was very low.

The interfacial concentration of Ce^{4+} increases rapidly at an applied $\Delta_o^w\phi \geq 0.2$ V (as shown in Figure 2b) and, as EDOT is a neutral molecule, the oxidant:monomer ratio also increases rapidly scanning positively. At +0.4 V for example, the Ce^{4+}:EDOT ratio may be as large as 400:1 (*i.e.,* 2 M Ce^{4+} and 5 mM EDOT). Thus, at 1 $mV{\cdot}s^{-1}$, the relatively long contact time with the large excess of interfacial Ce^{4+} may deplete the interfacial concentration of EDOT monomers and overoxidise oligomers present in the organic diffuse layer. These overoxidised oligomers may then be incapable of forming longer chains during subsequent CV cycles to reach the critical chain length. Electrochemical evidence of overoxidation was the absence of a steady build-up of charge with successive cycles (see cycles 5 to 9 in Figure S13d), meaning that the electroactivity of the adsorbed species was very low. Direct visual evidence of overoxidation was the coating of the L|L interface with a white precipitate (Figure S14). The latter redissolved upon agitating the liquid|liquid electrochemical cell.

As a general trend, the appearance of a nucleation loop is more likely as the interfacial concentration of Ce^{4+} increases and scan rate decreases. Thus, observing nucleation loops is favoured as the bulk aqueous concentration of Ce^{4+} increases, for example with a loop seen with 4 mM but not with 2 mM Ce^{4+} at 100 $mV{\cdot}s^{-1}$ (Figures S9 and S10), and as the positive edge of the PPW is extended. Additionally, as the scan rate increases, the appearance of the nucleation loop occurs during progressively later cycles, *i.e.,* during cycles 1, 2 and 7 for experiments at 1 (Figure S13a), 5 (Figure 6), and 100 $mV{\cdot}s^{-1}$ (Figure S10b), respectively. A systematic overview of the changes of the physical properties of PEDOT thin films formed as a function of scan rate will be carried out in a future study.

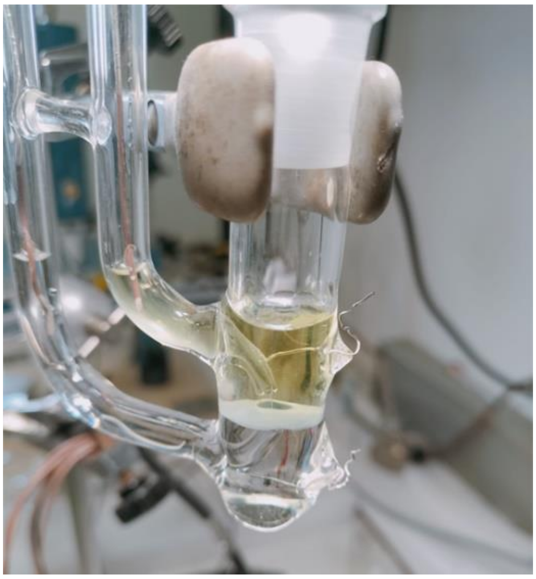

Figure S14. Image of the white precipitate formed after 15 cycles with 6 mM Ce^{4+} at 1 $mV \cdot s^{-1}$ as described in Figures S12b and S13. The white precipitate redissolves upon agitating the 4-electrode liquid|liquid electrochemical cell.

Supplementary References

(1) Smirnov, E.; Peljo, P.; Scanlon, M. D.; Girault, H. H. Gold Nanofilm Redox Catalysis for Oxygen Reduction at Soft Interfaces. *Electrochim. Acta* **2016**, *197*, 362–373.

(2) Kudoh, Y.; Akami, K.; Matsuya, Y. Chemical Polymerization of 3,4-Ethylenedioxythiophene Using an Aqueous Medium Containing an Anionic Surfactant. *Syn. Met.* **1998**, *98* (1), 65–70.

(3) Molina-Osorio, A. F.; Yamamoto, S.; Robayo-Molina, I.; Gamero-Quijano, A.; Nagatani, H.; Scanlon, M. D. A Soft on/off Switch Based on the Electrochemically Reversible H–J Interconversion of a Floating Porphyrin Membrane. *Chem. Sci.* **2021**, *12* (30), 10227–10232.

(4) Quiroga, M. V. C.; Benavidez, T.; Yudi, L. M.; Baruzzi, A. M.; Vogelsang, M.; Jones, H.; Santos, E. Evanescent-Wave Cavity Ring-down Spectroscopy Applied to Electrochemical Ion Transfer at Liquid–Liquid Interfaces. *Electrochem. Commun.* **2012**, *23*, 1–4.

(5) Gamero-Quijano, A.; Herzog, G.; Scanlon, M. D. Bioelectrochemistry of Cytochrome c in a Closed Bipolar Electrochemical Cell. *Electrochem. Commun.* **2019**, *109*, 106600.

(6) Samec, Z. Electrochemistry at the Interface between Two Immiscible Electrolyte Solutions (IUPAC Technical Report). *Pure Appl. Chem.* **2004**, *76* (12), 2147–2180.

(7) Lehane, R. A.; Gamero-Quijano, A.; Malijauskaite, S.; Holzinger, A.; Conroy, M.; Laffir, F.; Kumar, A.; Bangert, U.; McGourty, K.; Scanlon, M. D. Electrosynthesis of Biocompatible Free-Standing PEDOT Thin Films at a Polarized Liquid|Liquid Interface. *J. Am. Chem. Soc.* **2022**, *144* (11), 4853–4862.

(8) Gamero-Quijano, A.; Manzanares, J. A.; Ghazvini, S. M. B. H.; Low, P. J.; Scanlon, M. D. Potential-Modulated Ion Distributions in the Back-to-Back Electrical Double Layers at a Polarised Liquid|Liquid Interface Regulate the Kinetics of Interfacial Electron Transfer. *ChemElectroChem* **2023**, *10* (3), e202201042.

(9) Borukhov, I.; Andelman, D.; Orland, H. Steric Effects in Electrolytes: A Modified Poisson-Boltzmann Equation. *Phys. Rev. Lett.* **1997**, *79* (3), 435–438.

(10) Cervera, J.; García-Morales, V.; Pellicer, J. Ion Size Effects on the Electrokinetic Flow in Nanoporous Membranes Caused by Concentration Gradients. *J. Phys. Chem. B* **2003**, *107* (33), 8300–8309.

(11) Cervera, J.; Ramírez, P.; Manzanares, J. A.; Mafé, S. Incorporating Ionic Size in the Transport Equations for Charged Nanopores. *Microfluid. Nanofluid.* **2010**, *9* (1), 41–53.

(12) Bendrea, A.-D.; Cianga, L.; Ailiesei, G.-L.; Ursu, E.-L.; Göen Colak, D.; Cianga, I. 3,4-Ethylenedioxythiophene (EDOT) End-Group Functionalized Poly-ε-Caprolactone (PCL): Self-Assembly in Organic Solvents and Its Coincidentally Observed Peculiar Behavior in Thin Film and Protonated Media. *Polymers* **2021**, *13* (16), 2720.

(13) Kontturi, A. K.; Kontturi, K.; Manzanares, J. A.; Mafé, S.; Murtomäki, L. Ion Pairing in the Analysis of Voltammetric Data at the ITIES: RbTPB and RbTPBCl in 1,2-dichloroethane. *Ber. Bunsenges. Phys. Chem.* **1995**, *99* (9), 1131–1136.

(14) Li, F.; Zhou, C.; Klinkova, A. Simulating Electric Field and Current Density in Nanostructured Electrocatalysts. *Phys. Chem. Chem. Phys.* **2022**, *24* (42), 25695–25719.

(15) Ahonen, H. J.; Lukkari, J.; Kankare, J. N- and p-Doped Poly(3,4-Ethylenedioxythiophene): Two Electronically Conducting States of the Polymer. *Macromolecules* **2000**, *33* (18), 6787–6793.

(16) Hillman, A. R.; Daisley, S. J.; Bruckenstein, S. Ion and Solvent Transfers and Trapping Phenomena during N-Doping of PEDOT Films. *Electrochim. Acta* **2008**, *53* (11), 3763–3771.

(17) Chen, X.; Inganäs, O. Three-Step Redox in Polythiophenes: Evidence from Electrochemistry at an Ultramicroelectrode. *J. Phys. Chem.* **1996**, *100* (37), 15202–15206.
(18) Łapkowski, M.; Pron, A. Electrochemical Oxidation of Poly(3,4-Ethylenedioxythiophene) — "in Situ" Conductivity and Spectroscopic Investigations. *Syn. Met.* **2000**, *110* (1), 79–83.
(19) Garreau, S.; Duvail, J. L.; Louarn, G. Spectroelectrochemical Studies of Poly(3,4-Ethylenedioxythiophene) in Aqueous Medium. *Syn. Met.* **2001**, *125* (3), 325–329.
(20) Damlin, P.; Kvarnström, C.; Ivaska, A. Electrochemical Synthesis and in Situ Spectroelectrochemical Characterization of Poly(3,4-Ethylenedioxythiophene) (PEDOT) in Room Temperature Ionic Liquids. *J. Electroanal. Chem.* **2004**, *570* (1), 113–122.
(21) Hossein-Babaei, F.; Chegini, E. The Complex Permittivity of PEDOT:PSS. *J. Chem. Phys.* **2023**, *158* (19), 194904.
(22) Lin, Y.; Zhao, Y.; Xin, Q.; Jiang, C.; Song, A. Electrical Control of the Optical Dielectric Properties of PEDOT:PSS Thin Films. *Opt. Mater.* **2020**, *108*, 110435.
(23) Du, Y.; Cui, X.; Li, L.; Tian, H.; Yu, W.; Zhou, Z. Dielectric Properties of DMSO-Doped-PEDOT:PSS at THz Frequencies. *Phys. Status Solidi B* **2018**, *255* (4), 1700547.
(24) Pasha, A.; Roy, A. S.; Murugendrappa, M. V.; Al-Hartomy, O. A.; Khasim, S. Conductivity and Dielectric Properties of PEDOT-PSS Doped DMSO Nano Composite Thin Films. *J. Mater. Sci: Mater. Electron.* **2016**, *27* (8), 8332–8339.
(25) Sahalianov, I.; Singh, S. K.; Tybrandt, K.; Berggren, M.; Zozoulenko, I. The Intrinsic Volumetric Capacitance of Conducting Polymers: Pseudo-Capacitors or Double-Layer Supercapacitors? *RSC Adv.* **2019**, *9* (72), 42498–42508.
(26) Volkov, A. V.; Wijeratne, K.; Mitraka, E.; Ail, U.; Zhao, D.; Tybrandt, K.; Andreasen, J. W.; Berggren, M.; Crispin, X.; Zozoulenko, I. V. Understanding the Capacitance of PEDOT:PSS. *Adv. Funct. Mater.* **2017**, *27* (28), 1700329.
(27) Tybrandt, K.; Zozoulenko, I. V.; Berggren, M. Chemical Potential–Electric Double Layer Coupling in Conjugated Polymer–Polyelectrolyte Blends. *Sci. Adv.* **2017**, *3* (12), eaao3659.
(28) Proctor, C. M.; Rivnay, J.; Malliaras, G. G. Understanding Volumetric Capacitance in Conducting Polymers. *J. Polym. Sci. B Polym. Phys.* **2016**, *54* (15), 1433–1436.
(29) Sahalianov, I.; Say, M. G.; Abdullaeva, O. S.; Ahmed, F.; Glowacki, E.; Engquist, I.; Berggren, M.; Zozoulenko, I. Volumetric Double-Layer Charge Storage in Composites Based on Conducting Polymer PEDOT and Cellulose. *ACS Appl. Energy Mater.* **2021**, *4* (8), 8629–8640.
(30) Girault, H. H. Section 1.2.2: The Fermi-Dirac Distribution. In *Analytical and Physical Electrochemistry*; EPFL Press: New York, 2004; pp 25–26.
